# Conserved and divergent modes of substrate interaction define selective localizations and functions of a Cdc14 phosphatase

**DOI:** 10.1091/mbc.E26-04-0156

**Published:** 2026-09-25

**Authors:** Jun-Song Chen, Alaina H. Willet, Lesley A. Turner, Kathleen L. Gould

**Affiliations:** ^a^Department of Cell and Developmental Biology, Vanderbilt University School of Medicine, Nashville, TN 37232; University of California, Santa Cruz

## Abstract

Cdc14 phosphatases share conserved catalytic domains and enzymatic mechanisms. Despite functioning in disparate biological processes across eukaryotes, Cdc14 enzymes localize to analogous cellular structures from yeast to human cells. It remains unclear, however, whether modes of substrate recognition and intracellular targeting mechanisms are conserved among Cdc14 orthologs. Here, we address these questions using Clp1, the *Schizosaccharomyces pombe* Cdc14 phosphatase. We show that Clp1 utilizes a conserved hydrophobic pocket, originally defined in *Saccharomyces cerevisiae* Cdc14, to engage a subset of interactors, although unlike many *S. cerevisiae* Cdc14 substrates, these targets show a broad hydrophobic interaction motif spectrum. Disruption of the hydrophobic pocket selectively abrogates certain Clp1 interactions, localizations, and functions, while others are retained. Moreover, we find that nucleolar localization depends upon both hydrophobic pocket-mediated interactions and the Clp1 non-catalytic C-terminus. Our results suggest that Cdc14 uses multiple mechanisms to engage substrates and achieve proper spatial distribution for its diverse functions.

## Abstract (executive summary)

- Cdc14 phosphatases play important, but differing, roles across eukaryotic organisms. Whether Cdc14’s mode of substrate interaction is evolutionarily conserved is not known.
- The authors used mutational analysis, live-cell imaging, and structural modeling to show that disruption of a conserved hydrophobic pocket in the *S. pombe* Cdc14 enzyme selectively impairs specific protein interactions, subcellular localizations, and functions, while leaving others intact.
- The findings, which also implicate the non-catalytic C-termini in nucleolar targeting, suggest that Cdc14 phosphatases use multiple mechanisms, not yet all defined, to engage substrates and achieve proper spatial distribution for diverse functions.

## INTRODUCTION

Cdc14 family phosphatases are highly conserved from yeast to human (reviewed in Stegmeier and Amon, 2004; Mocciaro and Schiebel, 2010; Partscht and Schiebel, 2023). First characterized in yeast where they reverse mitotic cyclin-dependent kinase 1 (Cdk1) phosphorylation events and drive mitotic exit (reviewed in Trautmann and McCollum, 2002; Stegmeier and Amon, 2004; Clifford *et al.*, 2008a; Mocciaro and Schiebel, 2010), the Cdc14 paralogs Cdc14A and Cdc14B in higher eukaryotes are not required for mitotic progression (Berdougo *et al.*, 2008; Mocciaro *et al.*, 2010). Instead, they participate in an array of cellular processes including DNA repair, ciliogenesis, and gene expression control at both the transcriptional and translational levels (Clement *et al.*, 2011, 2012; Imtiaz *et al.*, 2018; Uddin *et al.*, 2019; Dietachmayr *et al.*, 2020; Partscht *et al.*, 2021; Partscht and Schiebel, 2023; Villarroya-Beltri *et al.*, 2023).

Despite playing important roles in disparate biological processes across eukaryotes, how Cdc14 engages its substrates has been investigated only in *Saccharomyces cerevisiae,* where it was found that short linear interaction motifs (SLiMs) containing a PxL consensus in substrates bind a highly conserved surface-exposed hydrophobic pocket within the Cdc14 catalytic domain (Kataria *et al.*, 2018). Whether other Cdc14 enzymes interact with their scaffolds and substrates similarly to *S. cerevisiae* Cdc14 is not known, but the fact that only a minority (6%–7%) of identified Cdc14 phosphatase substrates in *Schizosaccharomyces pombe* contain a sequence matching the PxL consensus motif (Zeisner *et al.*, 2025; Carme *et al.*, 2026) raises the possibility that alternative recognition mechanisms exist. Moreover, the importance of the hydrophobic pocket binding interface to Cdc14 enzyme localization and function in cells has not been determined in any organism.

Notably, although involvement in cell cycle processes varies across organisms, Cdc14 enzymes localize to overlapping cellular structures in a cell cycle-dependent manner in both yeast and multicellular eukaryotes (Bembenek and Yu, 2001; Cueille *et al.*, 2001; Trautmann *et al.*, 2001; Mailand *et al.*, 2002; Stegmeier and Amon, 2004; Trautmann and McCollum, 2005; Berdougo *et al.*, 2008). In both *S. pombe* and *S. cerevisiae*, they localize to the nucleolus and spindle pole bodies (SPBs) during interphase and to the cell division site, kinetochores, and mitotic spindle during mitosis (Trautmann and McCollum, 2002; Stegmeier and Amon, 2004; Clifford *et al.*, 2008a; Mocciaro and Schiebel, 2010). Similarly, mammalian Cdc14A and Cdc14B localize to centrosomes and the nucleolus, respectively, during interphase and to the cytoplasm and spindle (only Cdc14A) during mitosis (Bembenek and Yu, 2001; Mailand *et al.*, 2002; Berdougo *et al.*, 2008). Cdc14 proteins are thought to be sequestered away from their substrates when in the nucleolus and therefore, exit from the nucleolus during mitosis is considered a major activation step (Shou *et al.*, 1999; Visintin *et al.*, 1999; Mishra *et al.*, 2005; Chen *et al.*, 2008).

Here, we used the *S. pombe* Cdc14 phosphatase, Clp1, to define substrate interaction determinants and assess their evolutionary conservation. Clp1, unlike *S. cerevisiae* Cdc14, is not essential (Cueille *et al.*, 2001; Trautmann *et al.*, 2001), and therefore, we were able to address how substrate docking contributes to phosphatase localization and function. We found that mutationally disrupting the hydrophobic pocket in *S. pombe* Clp1 led to a variety of consequences for the enzyme. While it abrogated association with a cohort of binding partners, it left others intact, as judged by retention of a subset of Clp1 localizations and its function in stabilizing the cytokinetic ring (CR) against perturbation. In terms of potential motifs recognized by the conserved Clp1 hydrophobic pocket, we did not detect the previously defined PxL motif among them but rather a broader spectrum of hydrophobic sequences. We further demonstrate separable contributions of hydrophobic pocket-mediated docking and the non-catalytic C-terminus to Clp1 nucleolar targeting. Overall, our study indicates that Cdc14 phosphatases use multiple mechanisms to achieve proper cellular distribution and functions.

## RESULTS

### Mid1 binds the Clp1 catalytic domain through a conserved hydrophobic residue

We first examined the interaction between Clp1 and the anillin-like protein Mid1, which positions the CR centrally (reviewed in Rincon and Paoletti, 2012), and recruits Clp1 to this subcellular location (Clifford *et al.*, 2008b). Clp1’s CR localization and dephosphorylation of CR components, including the F-BAR protein Cdc15, paxillin-like Pxl1, and formin Cdc12, depend on Clp1 binding to Mid1 residues 431–481 (Clifford *et al.*, 2008b; Willet *et al.*, 2018; Mangione *et al.*, 2021). Here, we found that Clp1(1–372), which lacks the C-terminal non-catalytic domain, localized similarly to full-length Clp1 at CRs (Figure 1A), indicating that the Clp1 catalytic domain docks the enzyme to Mid1. To define more precisely what residues within Mid1 are necessary for Clp1 interaction, we performed alanine-scanning mutagenesis of GST-Mid1(431–481). Mutation of residues D447, L448, R449, and H450 abolished binding to MBP-Clp1(1–371) in vitro, whereas mutation of other amino acid clusters had no effect (Supplemental Figure S1, A–C). Individual amino acid substitutions revealed that the L448A and H450A mutants significantly reduced Mid1-Clp1 interaction, with the L448A mutation having the most severe effect (Supplemental Figure S1D).

**FIGURE 1:**
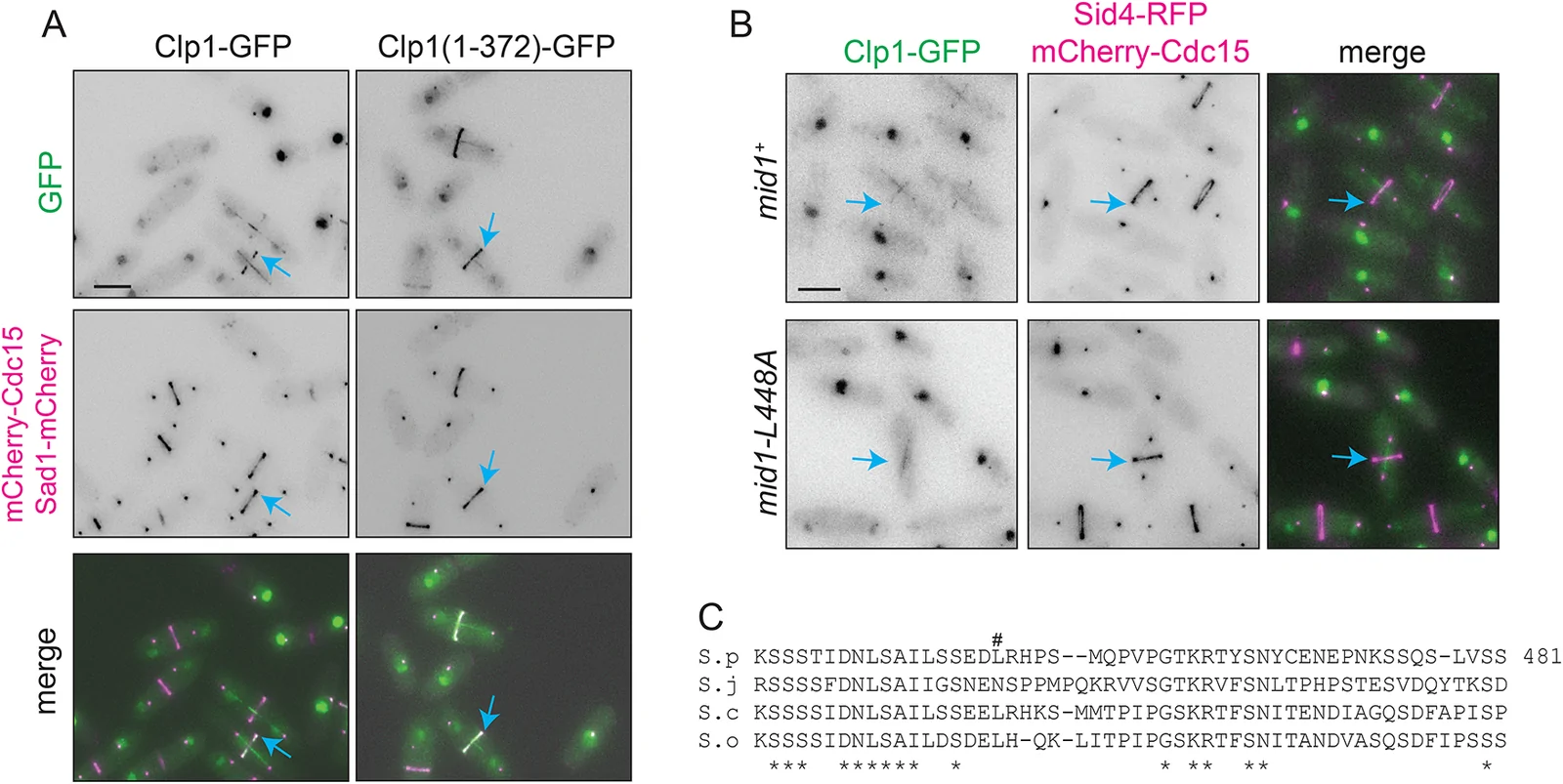
Characterization of the Mid1-Clp1 interaction. A and B) Representative live-cell images of the indicated strains grown at 25°C in YE. Blue arrows in A and B indicate the positions of fully formed CRs that have not yet begun constriction. Scale bar, 5 µm. C) Sequence alignment of four *Schizosaccharomyces* Mid1 proteins generated using Clustal Omega (Sievers and Higgins, 2021; Harris *et al.*, 2022). L448 is indicated with a “#.” Asterisks indicate identical residues within the *S. pombe* (S.p) 431–481 fragment. *S. japonicus* (S.j), *S. cryophilus* (S.c) and *S. octosporus* (S.o).

To test the importance of this binding interface in cells, we constructed a *mid1-L448A* strain. These cells formed centered septa, and Mid1-L448A-mNG localized normally (Supplemental Figure S1E), indicating that the mutation did not disrupt Mid1 function. However, Clp1-GFP was absent from the CRs of *mid1-L448A* cells (38 of 38 cells with CRs), although it localized normally to the mitotic spindle, kinetochores, SPBs, and nucleolus (Figure 1B). L448 is conserved among Mid1 orthologs but is not part of a PxL motif, and there is no PxL motif within Mid1 residues 431–481 (Figure 1C). Thus, Mid1 engages Clp1 through a hydrophobic residue distinct from the PxL consensus defined for *S. cerevisiae* Cdc14 substrates (Kataria *et al.*, 2018).

### Nsk1 engages Clp1 via F168 within a conserved hydrophobic segment

Although Clp1 binds Mid1, it is not known to dephosphorylate it (Clifford *et al.*, 2008b). We therefore examined the binding parameters of a bonafide substrate, Nsk1, which emerged as the top hit in a proteomics screen identifying many Clp1 substrates (Chen *et al.*, 2013). Nsk1 co-localizes with Clp1 to the nucleolus during interphase and to the kinetochores during mitosis, where Nsk1 acts to ensure accurate chromosome segregation (Buttrick *et al.*, 2011; Chen *et al.*, 2011).

Two-hybrid and in vitro binding assays were used to define the region of Nsk1 interacting with the Clp1 catalytic domain as residues 151–182 (Figure 2A; Supplemental Figure S2, A and B). Mutation of either F167 or F168 within this Nsk1 fragment to alanine affected the ability of GST-Nsk1(151–182) to bind Clp1, with mutation of F167 reducing binding and mutation of F168 abrogating it; the F168A mutation also eliminated interaction with Clp1 in the two-hybrid assay (Figure 2B; Supplemental Figure S2C).

**FIGURE 2:**
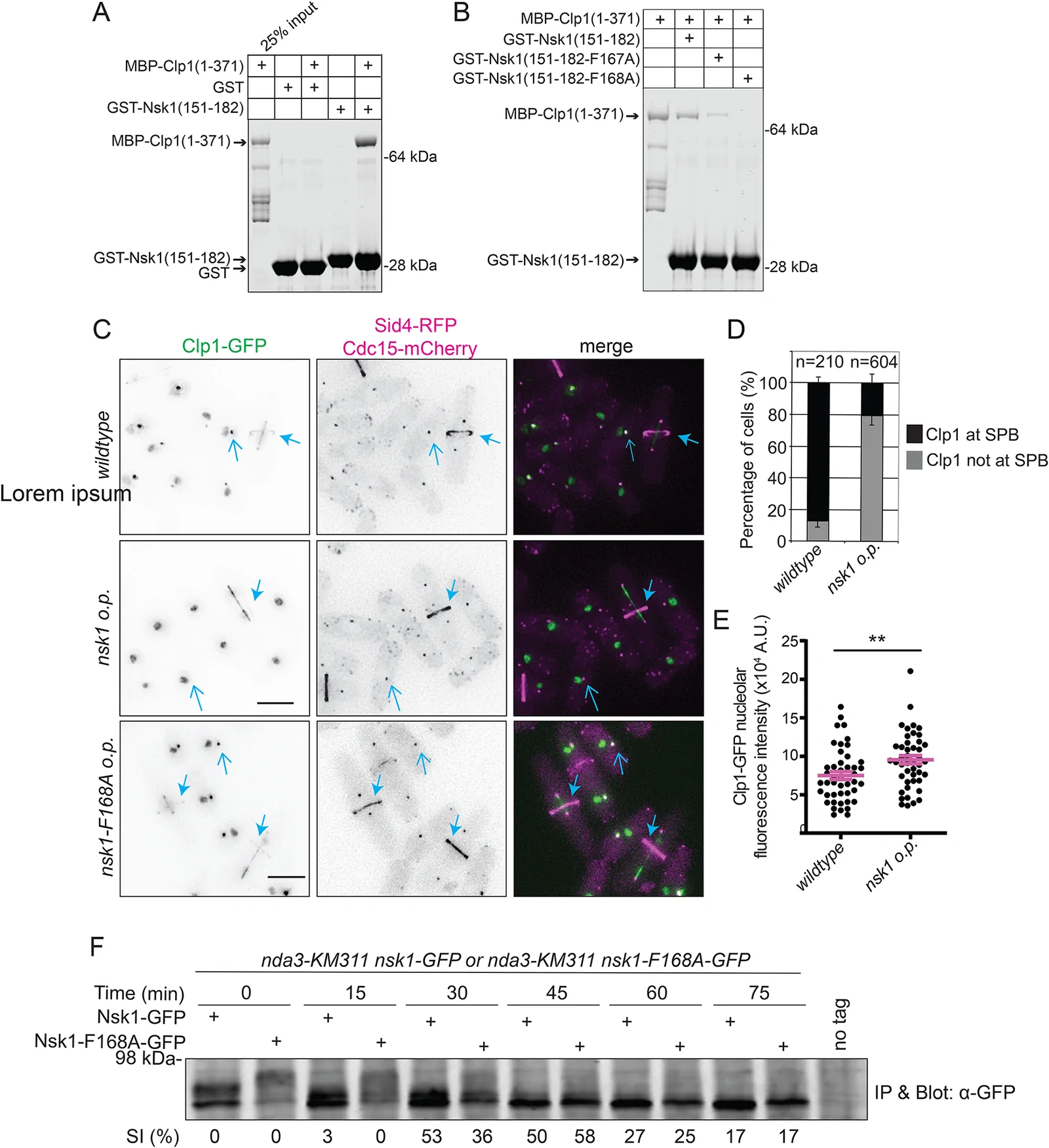
Characterization of the Nsk1-Clp1 interaction. A and B) Bead-bound recombinant GST, GST-Nsk1(151–182), or GST-Nsk1(151*–*182) was incubated with MBP-Clp1(1–371). Input was 25% of the amount used in the binding reaction. After washing, bound proteins were resolved by SDS–PAGE and stained with Coomassie blue. Molecular mass markers are to the right of the gels. C) Representative live-cell images of *clp1-GFP sid4-RFP cdc15-mCherry* cells grown at 32°C with uninduced *nsk1^+^* as well as *nsk1^+^* or *nsk1-F168A* over-expressed for 24 h. The closed arrows indicate examples of CRs marked by Cdc15-mCherry. Open arrows indicate SPBs marked by Sid4-RFP. Scale bar, 5 µm. D) Quantification of the percent of cells with Clp1 at an SPB from cells as in Supplemental Figure S2E. Error bars represent SD from three biological replicates. *n* = 210 cells for uninduced *nsk1^+^* expression (wildtype) and *n* = 604 cells for *nsk1* overexpression (o.p.). E) Quantification of the fluorescence intensity of Clp1 in the nucleolus. *n* = 45 cells of each condition from three biological replicates. Error bars represent SEM. ** *p* ≤ 0.01 (unpaired two-tailed Student's *t* test). F) Nsk1-GFP or Nsk1-F168A-GFP was immunoprecipitated (IP) from *nda3-KM311* cells blocked at 19°C, released for the indicated times, and detected by immunoblotting. SI, septation index.

We next wanted to obtain evidence that Nsk1-F168A no longer binds Clp1 in vivo. For this, we used the observation that overproduced Nsk1, which concentrated in the nucleolus during interphase and along the spindle during mitosis (Supplemental Figure S2D), altered the intracellular distribution of Clp1-GFP (Figure 2C). Consistent with competitive binding, Clp1 was no longer localized to CRs and was localized to an SPB in only ∼20% of cells overproducing Nsk1, in contrast to 90% of wild-type cells; Clp1 also became enriched in the nucleolus (Figure 2, C–E; Supplemental Figure S2E). Overexpression of Nsk1-F168A, in contrast, did not change Clp1 localization, indicating that Nsk1-F168A could no longer bind Clp1 and outcompete other Clp1 partners (Figure 2C), although Nsk1-F168A was produced to the same level and localized indistinguishably from wild-type Nsk1 (Supplemental Figure S2D). To provide evidence that the F168A mutation impacted Nsk1 function, we replaced *nsk1^+^* with *nsk1-F168A* at the endogenous locus and found it increased the sensitivity of *alp7∆* cells to the microtubule-destabilizing drug thiabendazole (Supplemental Figure S2F). Because Alp7 is involved in kinetochore-microtubule attachment (Tang and Toda, 2015), this result suggests that Nsk1-F168A has impaired kinetochore function, similar to *nsk1∆* as well as both phosphoablating and phosphomimetic *nsk1* mutants (Chen *et al.*, 2011). To determine if Nsk1-F168A was hyperphosphorylated relative to Nsk1, we performed a pro-metaphase block and release experiment using the ß-tubulin *nda3-KM311* mutant (Hiraoka *et al.*, 1984). We showed previously that the slowly migrating Nsk1 bands are due to phosphorylation, and while Clp1 participates in its dephosphorylation, other phosphatases ensure Nsk1’s complete dephosphorylation as cells progress through mitosis (Chen *et al.*, 2011). Consistent with abrogating the Clp1-Nsk1 interaction, Nsk1-F168A was more phosphorylated than Nsk1 at the arrest point and was dephosphorylated more slowly during mitotic progression (Figure 2F). However, the F168A mutant did not affect the Nsk1 localization pattern (Supplemental Figure S2G). F168 is conserved among *Schizosaccharomyces* Nsk1 orthologues and is within the most highly conserved region of Nsk1-related proteins (Supplemental Figure S2B). As with Mid1, this Nsk1 docking determinant extends beyond the PxL consensus described for *S. cerevisiae* Cdc14 substrates.

### Disruption of Clp1 docking to Cdc25 impairs mitotic timing

We next examined the binding parameters of another Clp1 substrate, the Cdc25 phosphatase (Esteban *et al.*, 2004; Wolfe and Gould, 2004; Zeisner *et al.*, 2025). Cdc25 activates Cdk1 and entry into mitosis by reversing inhibitory tyrosine phosphorylation on Cdk1 (reviewed in Nurse, 1990; Morgan, 1997). In a positive feedback loop, Cdc25 is itself activated by Cdk1-mediated phosphorylation (Pomerening *et al.*, 2003) and dephosphorylated at mitotic exit in a Clp1-dependent manner; Cdc25 remains hyperphosphorylated and more abundant in *clp1∆* cells (Esteban *et al.*, 2004; Wolfe and Gould, 2004). This effect on Cdc25 was presumed to underlie why *clp1∆* cells enter mitosis at a reduced cell size (Cueille *et al.*, 2001; Trautmann *et al.*, 2001; Esteban *et al.*, 2004; Wolfe and Gould, 2004).

We extended previous work (Wolfe and Gould, 2004) to define more precisely the Clp1-binding site within Cdc25. We validated a direct interaction of Clp1(1–371) with Cdc25(79–197) and narrowed down the interaction region within Cdc25 to residues 108–167 in vitro with recombinant proteins (Supplemental Figure S3, A and B). We used alanine-scanning mutagenesis of groups of amino acids conserved among *Schizosaccharomyces* Cdc25 proteins to identify a Cdc25 mutant incapable of binding Clp1. This analysis revealed that the F129A, R131A, P133A, Y137A (hereafter Cdc25-4A) and A123G, L124A, S127A mutants abolished interaction with Clp1 in vitro, whereas two other mutants did not (Supplemental Figure S3, A and C).

To test the importance of this binding interface to Cdc25 phosphostate and stabilization in vivo, we endogenously replaced *cdc25^+^* with *cdc25-4A,* and to facilitate detection, tagged it with Myc_13_. Cdc25-4A-Myc_13_ was hyperphosphorylated and more abundant compared with Cdc25-Myc_13_ (Figure 3A), confirming previous reports that Clp1 is a major phosphatase for Cdc25 at mitotic exit that controls its abundance (Esteban *et al.*, 2004; Wolfe and Gould, 2004). Cdc25-4A was, however, not phosphorylated to the same extent nor as abundant as Cdc25 from *clp1∆* cells (Figure 3A). We next overproduced Cdc25 or Cdc25-4A and assayed whether they disrupted Clp1 intracellular distribution. Overproduction of Cdc25 led to displacement of Clp1-GFP from CRs and the spindle in 29 of 33 cells, most likely by outcompeting other binding partners, whereas 39 of 41 cells retained CR and spindle localization of Clp1-GFP when Cdc25-4A was overproduced (Figure 3B). As a control, we detected 31 of 33 cells with Clp1-GFP at CRs and spindles with overproduction of the vector alone. This indicates that Cdc25-4A cannot stably engage Clp1. Consistent with the elevated phosphorylation and abundance of Cdc25-4A, *cdc25-4A* cells were shorter than wild-type but in accord with the relative phosphostate, not as short as *clp1∆* cells (Figure 3C).

**FIGURE 3:**
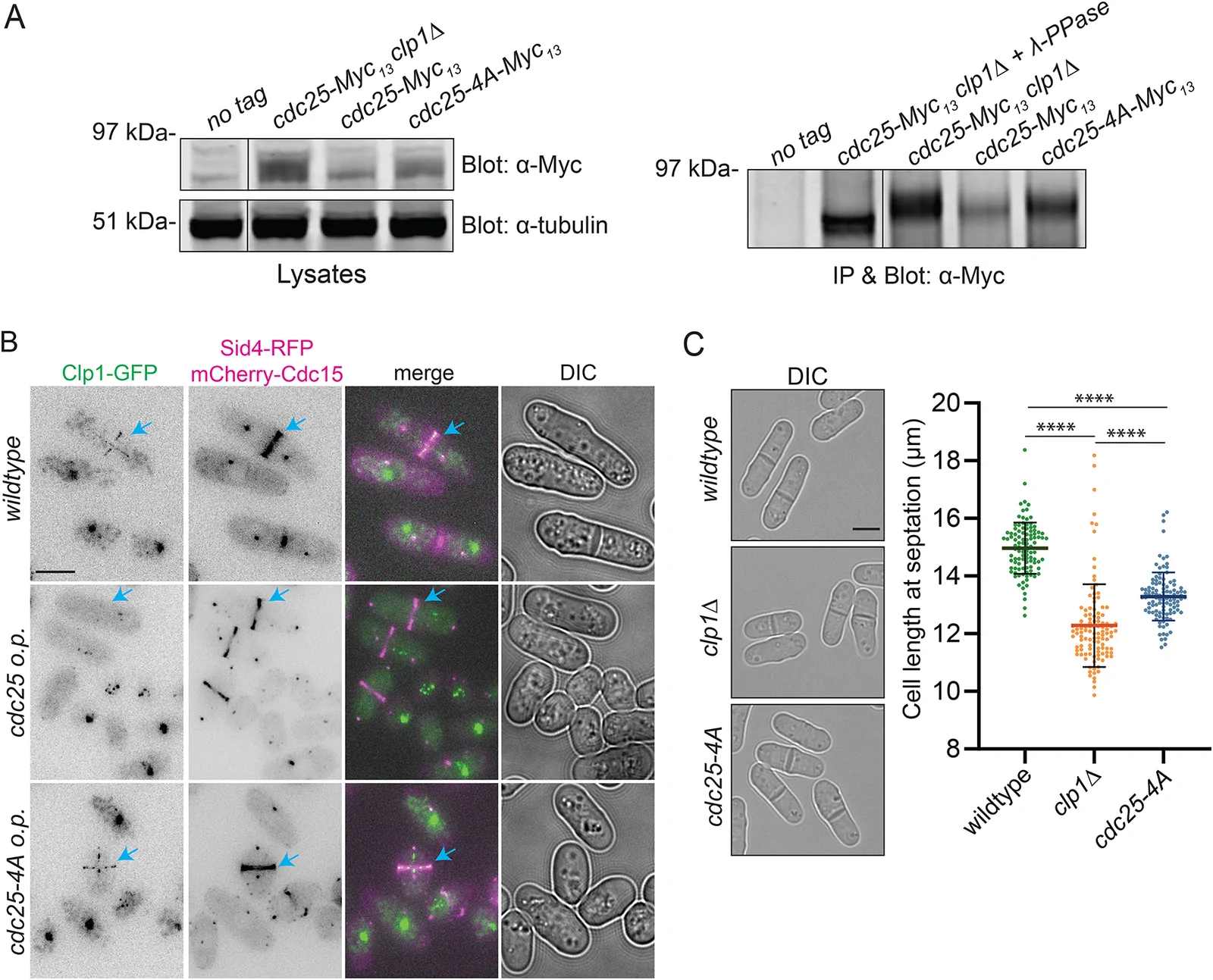
Characterization of the Clp1-Cdc25 interaction. A) Proteins were IPed from a portion of denatured lysates of the indicated strains with α-Myc antibody (9E10). The remainder of the lysates (left panel) and the IPs (right panel) were resolved by SDS–PAGE, transferred to a PVDF membrane, and blotted with anti-α-tubulin and/or α-Myc antibody (9E10). B) Representative live-cell images of the indicated strains containing empty vector (wildtype) or overexpressing *cdc25* or *cdc25-4A* for 20 h at 32°C. Blue arrows point to the position of CRs in anaphase B cells, defined by the presence of an unconstricted CR and two well-separated SPBs. C) Cell length at septation of the indicated strains grown at 32°C in YE. DIC: Differential Interference Contrast. *n* ≥ 103 cells per strain. Error bars represent SD. **** *p* < 0.0001 by one-way ANOVA.

Taken together, our data on Clp1-Cdc25 interaction provide a third example in which the Clp1 interaction motif within a partner is broader than the PxL consensus defined for *S. cerevisiae* Cdc14. Alignment of 10 amino acids around the necessary binding residues on the partner side of the three Clp1 interactions emphasized the variability in their sequence (Supplemental Figure S3D).

### Divergent substrates bind the conserved Clp1 hydrophobic pocket

Although the binding regions within Mid1, Nsk1, and Cdc25 varied, AlphaFold3 (AF; Abramson *et al.*, 2024) modeling predicted that each engages Clp1 residues Q111 and/or W113 (Figure 4A). Q111 and W113 are conserved among *Schizosaccharomyces* species and are orthologous to the hydrophobic pocket residues defined in *S. cerevisiae* Cdc14 as important for substrate interaction (Figure 4B; Kataria *et al.*, 2018). To confirm that these residues mediate partner interactions in *S. pombe*, we generated the same substitutions in MBP-Clp1(1-371), Q111L or W113R, previously shown to disrupt Cdc14 hydrophobic pocket-mediated interactions in *S. cerevisiae* (Kataria *et al.*, 2018). These Clp1 mutants were then tested for binding to fragments of Mid1, Nsk1, and Cdc25. Both mutations eliminated binding to all three proteins (Figure 4, C and D). Thus, hydrophobic residues within unrelated sequence contexts converge on a conserved catalytic docking surface.

**FIGURE 4:**
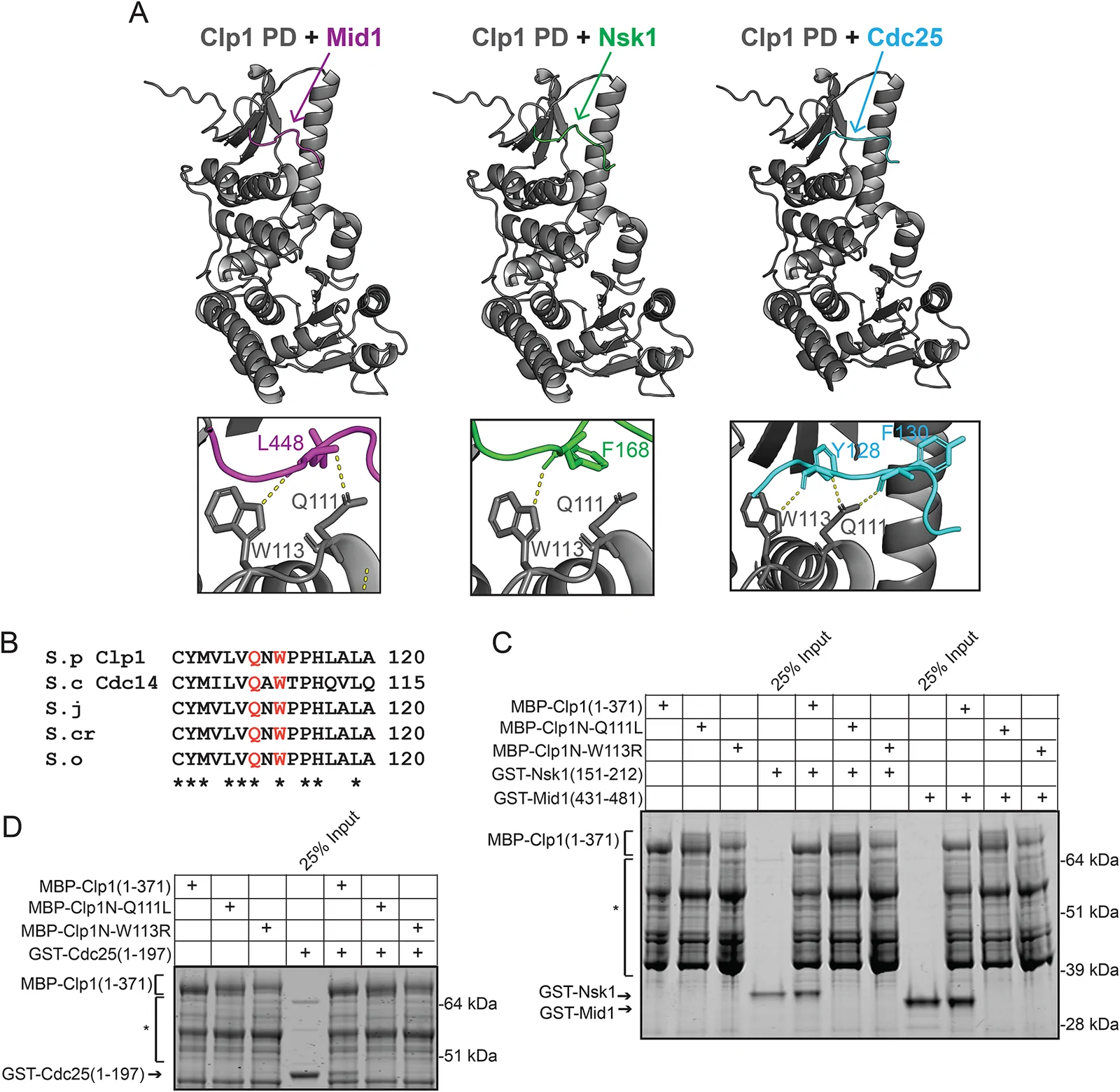
Clp1 binding partners interact with a conserved hydrophobic pocket in Clp1. A) AlphaFold3 (AF)-predicted structures of the Clp1-Nsk1, Clp1-Mid1, and Clp1-Cdc25 complexes. Mid1 residues 431–481 were modelled, and residues 444–451 are shown in magenta. Nsk1 residues 151–212 were modelled, and residues 164–172 are shown in green. Cdc25 residues 79–197 were modelled, and residues 84–90 are shown in cyan. Bottom insets show zoomed-in views of the AF-predicted interaction interfaces with side chain displays of interacting residues. PD, phosphatase domain. B) Sequence alignment of hydrophobic pocket residues in Cdc14 enzymes generated by Clustal Omega (Sievers and Higgins, 2021; Harris *et al.*, 2022). *S. pombe* (S.p), *S. cerevisiae* (S.c), *S. japonicus* (S.j), *S. cryophilus* (S.cr), *S. octosporus* (S.o). Identical amino acids are indicated with asterisks, and the number of the last amino acid in the motifs is to the right. Clp1 Q111 and W113 and orthologous residues are in orange. C and D) Bead-bound MBP-Clp1(1–371), MBP-Clp1(1-371)-Q111L, or MBP-Clp1(1-371)-W113R were incubated with GST-Nsk1(151–182), GST-Mid1(431–481), or GST-Cdc25(1–197). After washing, bound proteins were resolved by SDS–PAGE and visualized by staining with Coomassie blue. Molecular mass standards are to the right of the gels. Asterisks indicate MBP-Clp1 degradation products or co-purifying bacterial proteins.

### Hydrophobic pocket mutations selectively disrupt localizations and functions

To determine how disrupting hydrophobic pocket-mediated interactions affect Clp1 intracellular distribution and function, we generated *clp1-Q111L, clp1-W113R,* and *clp1-Q111L, W113R* alleles by replacing *clp1^+^* at the endogenous locus. *clp1∆* cells divide at a shorter cell size due to Clp1’s function in the regulation of mitotic entry (Cueille *et al.*, 2001; Trautmann *et al.*, 2001; Esteban *et al.*, 2004; Wolfe and Gould, 2004). Thus, we first measured each mutant's cell length at septation to ascertain its mitotic entry function. *clp1-Q111L*, *clp1-W113R,* and *clp1-Q111L, W113R* cells were shorter than wild-type cells, albeit not as short as two catalytically inactive mutants, *clp1-D257A* and *clp1-C286S* (Wolfe *et al.*, 2006), or *clp1∆* cells (Figure 5, A and B; Supplemental Figure S4A). Thus, the mutants partially lost Clp1’s mitotic control function.

**FIGURE 5:**
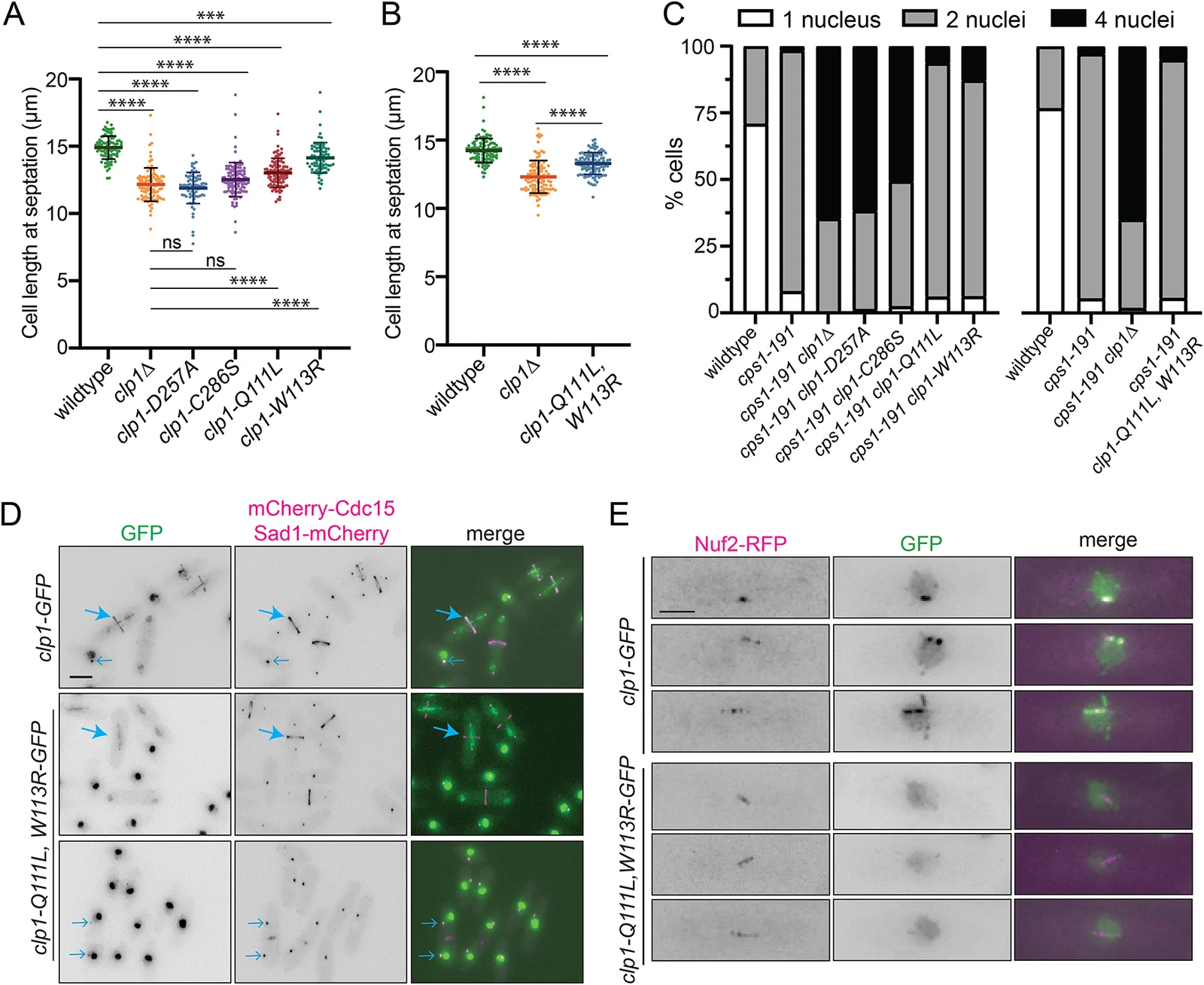
Characterization of *clp1* mutant strains. A and B) Cell length at septation of the indicated strains grown at 32°C. *n* ≥ 70 cells per strain. Error bars represent SD. **** *p* < 0.0001 and *** *p* < 0.001 by one-way ANOVA. C) Percent of cells containing the indicated number of nuclei for the indicated strains grown at 25°C and shifted to 36°C for 6 h before fixing and staining with DAPI and methyl blue. The left and right histogram panels were from separate experiments, each performed in duplicate, and the data were pooled. *n* > 200 cells per strain per experiment. D and E) Representative live-cell images of the indicated strains grown at 25°C in YE. Blue arrows indicate cells with (upper panels) or without (lower panels) SPB or CR localization of Clp1 proteins. Scale bars, 5 µm for D and 3 µm for E.

Another function of Clp1 is to stabilize the CR against perturbation and prevent entry into a second mitosis when cells fail septum assembly due to a mutation in the glucan synthase, Bgs1 (*cps1-191*; Le Goff *et al.*, 1999; Liu *et al.*, 2000; Mishra *et al.*, 2004). Interestingly, unlike the null and catalytically inactive mutants, *clp1-Q111L*, *clp1-W113R,* and *clp1-Q111L, W113R* cells functioned like wild-type in the *cps1-191* mutant, exhibiting no appreciable ploidy increase (Figure 5C). Also, the mutants were not sensitive to perturbation of the CR produced by a low concentration of latrunculin A, a reagent used to depolymerize F-actin in cells (Ayscough, 1998), as are *clp1∆, clp1-D257A,* and *clp1-C286S* cells (Supplemental Figure S4B). If anything, *clp1-Q111L, W113R* cells tolerated the drug better than wild-type (Supplemental Figure S4B). Thus, the QW hydrophobic pocket mutants retain Clp1’s function in CR stabilization.

To explore the reason for the differential functions retained by the hydrophobic pocket mutants, we examined their localizations and abundance. Strikingly, while the level of Clp1-Q111L, W113R was the same as wildtype (Supplemental Figure S4C), all three mutants were absent from the CR, the spindle midzone, and kinetochores (Figure 5, D and E; Supplemental Figure S5A). Specifically, Clp1- Q111L, W113R was not detected at CRs of 32 cells with CRs, at the spindle midzone of 32 cells proceeding through mitosis, or on kinetochores of 32 of 33 cells with defined kinetochores during mitosis. This result suggests that like Mid1, Nsk1, and Cdc25, Clp1 substrates pertinent to spindle midzone assembly and kinetochore function, such as the chromosomal passenger complex (CPC), kinesin-6 motor Klp9p, microtubule antiparallel bundler Ase1p, and the monopolin complex (Trautmann *et al.*, 2004; Bohnert *et al.*, 2009; Choi *et al.*, 2009; Fu *et al.*, 2009), are also unlikely to interact with Clp1 hydrophobic pocket mutants. In support of the loss of kinetochore function, like *clp1∆* (Trautmann *et al.*, 2004), *clp1-Q111L, W113R* was synthetically lethal with the CPC mutant, *cut17-275* (Supplemental Figure S5B).

The changes in localization of Clp1-Q111L, W113R to SPBs were more nuanced. Clp1-Q111L, W113R was observed at SPBs in late anaphase cells, cells with a septum, daughter cells that had just divided from one another, and short interphase cells. However, SPB localization was not detected in longer interphase cells (Figure 5D; Supplemental Figure S5A). Surprisingly, nucleolar localization seemed unaffected by the hydrophobic pocket mutations, assessed by line scans through the nucleus (Figure 5D; Supplemental Figure S5, C and D). Also, localization to anaphase spindle ends was preserved (Figure 5D). The combination of these results suggests that while certain binding interactions are mediated by the Q111 and W113 residues of the hydrophobic pocket, others are not.

Because the Q111 and W113 residues are not conserved in mammalian Cdc14 phosphatases, we tested whether mutating residues of the hydrophobic pocket conserved in human Cdc14 enzymes would eliminate Clp1 SPB, spindle, and nucleolar localizations. We changed Y106 (a strictly conserved residue central to the hydrophobic pocket) and M107 (also present in hCdc14B) to alanines alone or in combination with Q111L and W113R. Whereas we had found that Clp1-Q111L, W113R-GFP abundance was the same as Clp1-GFP (Supplemental Figure S4C), Clp1-Y106A, M107A-GFP and the quadruple mutant were significantly less abundant than Clp1-GFP (Supplemental Figure S6A), indicating that these mutations likely destabilized the hydrophobic pocket and hence, Clp1. Despite their lower abundance, the new mutants were still detected at SPBs, along the spindle, and in the nucleolus (Supplemental Figure S6B). While these data do not rule out the possibility that still other residues of the hydrophobic pocket engage independently with a subset of substrates, they also raise the possibility that some binding partners engage with Clp1 via alternative docking sites on the enzyme.

#### Nucleolar localization is maintained despite disruption of hydrophobic pocket-mediated docking

The retention of nucleolar localization by the Clp1 hydrophobic pocket mutants was unexpected because in *S. cerevisiae*, Net1 anchors Cdc14 in the nucleolus during interphase (Shou *et al.*, 1999; Visintin *et al.*, 1999; Traverso *et al.*, 2001), and the analogous Q and W mutations in Cdc14 disrupt Net1 interaction in vitro (Kataria *et al.*, 2018). We therefore examined nucleolar localization of Clp1 and Clp1-Q111L, W113R more carefully. Contrary to our assumption that the majority of interphase Clp1 is nucleolar, only ∼40% of wild-type Clp1 was in the interphase nucleolus (Figure 6, A and B). Although less than Clp1-GFP, still more than 30% of Clp1-Q111L, W113R-GFP was nucleolar (Figure 6, A and B). This finding prompted us to examine Clp1 nucleolar dynamics using fluorescence recovery after photobleaching (FRAP). We found that if a small region of the nucleolus was photobleached, Clp1-GFP recovered very rapidly (t_1/2_ = 3.0 s) and Clp1-Q111L, W113R-GFP recovered somewhat faster (t_1/2_ = 1.4 s; Figure 6, C and D). However, there was a significant immobile fraction for both proteins (Figure 6, C and D). When nearly the entire nucleolus was photobleached, there was rapid but very little recovery of either protein (Figure 6, C and D), similar to FRAP results for *S. cerevisiae* Cdc14 (Tomson *et al.*, 2009). These results suggest that parameters other than hydrophobic patch-mediated interactions contribute to Clp1 nucleolar localization.

**FIGURE 6:**
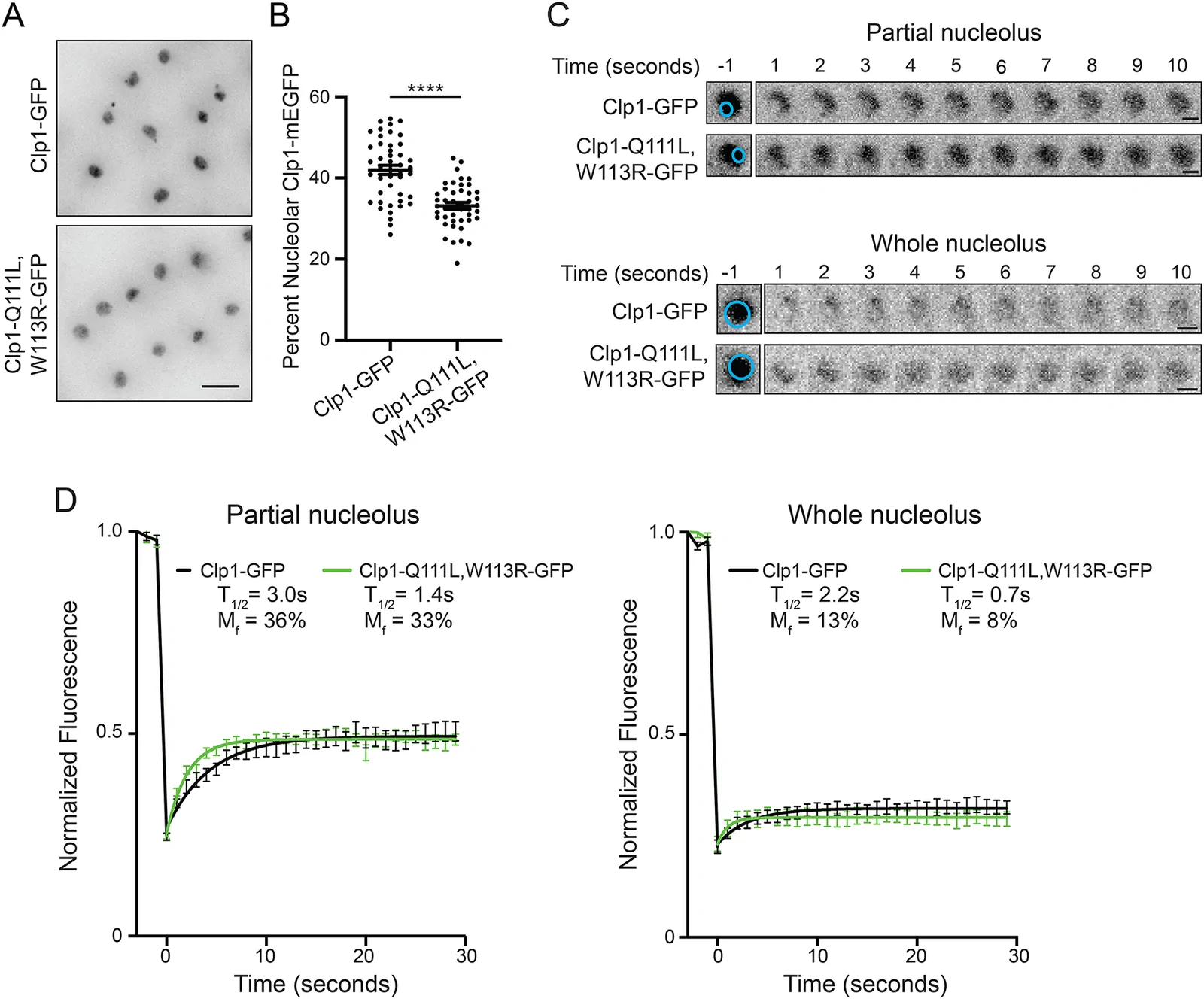
Characterization of Clp1 nucleolar dynamics. A) Representative live-cell images of interphase cells expressing Clp1-GFP or Clp1-Q111L,W113R-GFP. Scale bar, 5 µm. B) Quantification of the percent of Clp1-GFP signal that is nucleolar. *n* = 45 for both strains, and data are from three independent experiments. Error bars represent SEM. **** *p* < 0.0001 by Student's *t* test. C) Representative live-cell time-lapse images that are zoomed in on the nucleolus of cells expressing Clp1-GFP or Clp1-Q111L,W113R-GFP. GFP fluorescence was photobleached at time 0, and cells were imaged every 1s. Scale bars, 1 μm. D) Quantification of the fluorescence recovery after photobleaching of the strains from C. Error bars represent SEM. *n* = 15 for both strains for the left graph, and *n* = 8 for WT and *n* = 7 for the mutant in the right graph. Data are from three independent experiments. Clp1-GFP halftime (T_1/2_) and mobile fraction (M_f_) are indicated.

#### The Clp1 non-catalytic tail contributes to nucleolar targeting

To determine if the C-terminus plays a role in targeting Cdc14 phosphatases to the nucleolus, we truncated Clp1-Q111L, W113R, so that only the catalytic domain was produced. Cdc14 catalytic domains no longer contain a nuclear localization sequence (NLS; Mohl *et al.*, 2009; Chen *et al.*, 2013; Figure 7A) but are sufficiently small to access the nucleus (e.g., Figure 1A). Although expressed at wild-type levels (Figure 7B), Clp1(1-372)-Q111L, W113R-GFP exhibited diffuse nuclear distribution with greatly reduced nucleolar enrichment (Figure 7, C–E). The difference was best appreciated by examining single Z-stack images and performing line scans through the nucleus (Figure 7, D and E). These findings indicate that robust Clp1 nucleolar targeting is multimodal, requiring both hydrophobic pocket-mediated docking and the C-terminus. Finally, we tested whether the truncation mutants supported Clp1’s role in CR stabilization. Their ability to stabilize the CR against perturbation was intact, confirming that this function is independent of nucleolar localization, as well as the hydrophobic pocket and the C-terminus (Figure 7F).

**FIGURE 7:**
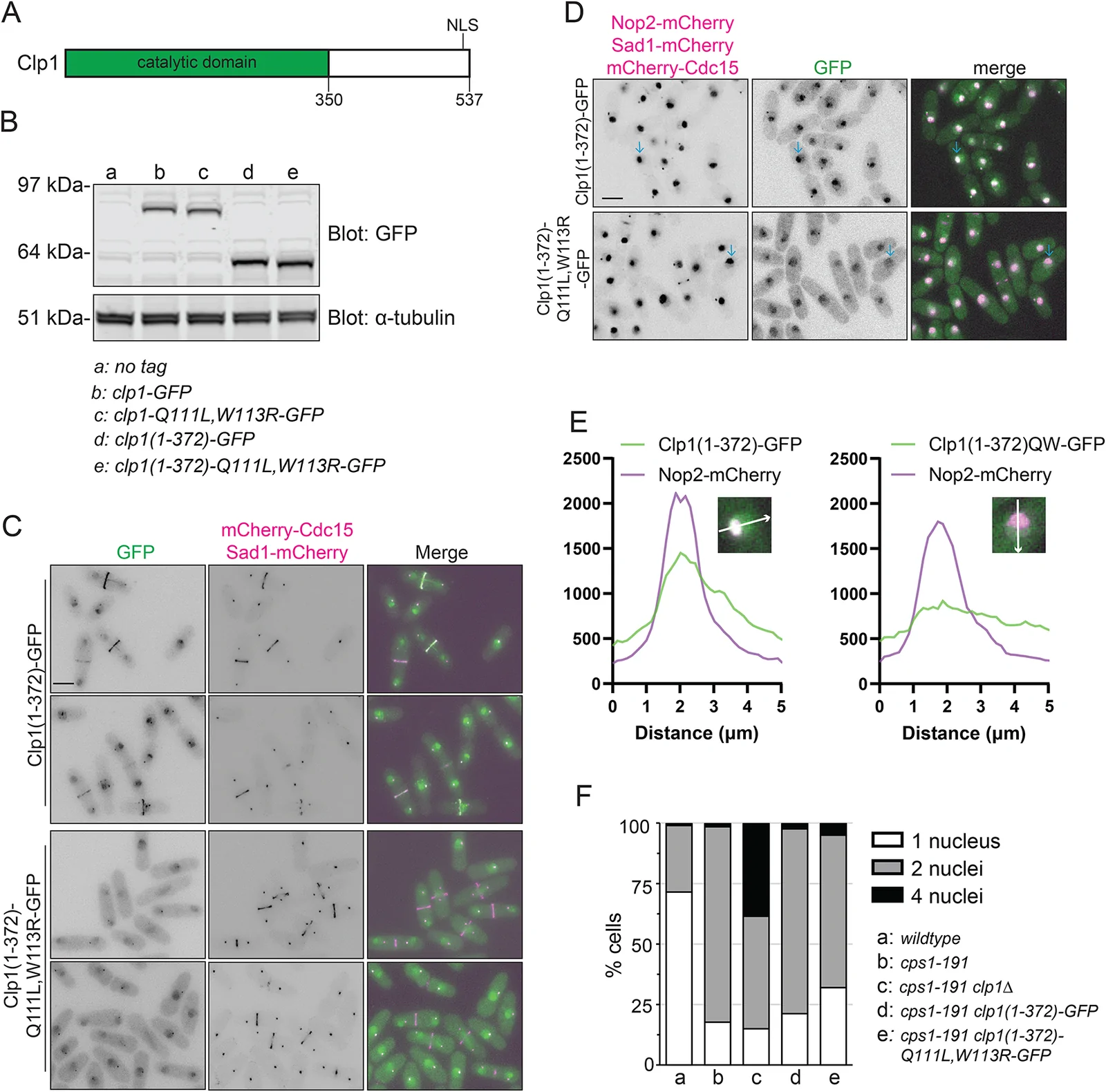
The Clp1 non-catalytic tail contributes to nucleolar targeting. A) A schematic of Clp1, drawn to scale, with the catalytic domain in green and the NLS position indicated. B) Denatured lysates of the indicated strains were immunoblotted with anti-α-tubulin and anti-GFP antibodies. C and D) Representative live-cell images of the indicated strains grown at 25°C in YE. Blue arrows in D indicate examples of cells with (upper panels) or without (lower panels) nucleolar enrichment of Clp1 proteins. Scale bars, 5 µm. E) Plots of representative line scans across the nuclei of cells indicated with blue arrows in D. Zoomed-in views of the nuclei are shown. White arrows are 5 µm in length and denote the position and direction of the line scans. QW = Q111L,W113R. F) Percent of cells containing the indicated number of nuclei for the indicated strains grown at 25°C and shifted to 36°C for 6 h before fixing and staining with DAPI and methyl blue. *n* > 200 cells per strain from one of the two experiments with similar results.

## DISCUSSION

Despite their involvement in disparate biological functions across eukaryotic organisms, Cdc14 phosphatases exhibit remarkably conserved sequences, catalytic mechanisms, and intracellular distributions (Wang *et al.*, 2004; Mocciaro and Schiebel, 2010; Bremmer *et al.*, 2012; Li *et al.*, 2015). Here, we provide new insight into the functional conservation of a substrate-binding hydrophobic pocket within the catalytic domain and a docking motif within substrates, as defined in *S. cerevisiae* (Kataria *et al.*, 2018). We show that certain binding interactions, as well as some targeting cues and functions, are solely mediated by certain residues of the *S. pombe* Cdc14 phosphatase hydrophobic pocket. However, other Clp1 localizations and functions are independent of this interaction mode, and we identify a key role for the C-terminus in nucleolar targeting. Together, our findings indicate that Cdc14 phosphatases engage their partners through both conserved and divergent mechanisms, and this modularity might enable their functional diversification.

Proteomic screens using catalytically inactive substrate-trapping mutants, along with quantitative mass spectrometry studies, have identified dozens of substrates for *S. pombe* Clp1 and *S. cerevisiae* Cdc14 (Bloom and Cross, 2007; Chen *et al.*, 2013; Kao *et al.*, 2014; Zeisner *et al.*, 2025). Many *S. cerevisiae* Cdc14 substrates harbor a PxL consensus motif (Kataria *et al.*, 2018). In contrast, none of the three Clp1 interactors examined here depended on a PxL consensus for association, consistent with bioinformatic analysis indicating that only 6%–7% of known Clp1 substrates contain this sequence (Zeisner *et al.*, 2025; Carme *et al.*, 2026). We also did not identify an alternative binding motif. AF models, however, underscored the commonality of hydrophobic amino acids engaging the Clp1 hydrophobic pocket, suggesting a broadly conserved but sequence-diverse mode of interaction typical of SLiMs (Tompa *et al.*, 2014). While disrupting or adding a hydrophobic pocket-mediated interaction in a substrate changed its dephosphorylation timing during the *S. cerevisiae* cell cycle (Kataria *et al.*, 2018), disrupting this interaction mode in *S. pombe* led to a much broader range of consequences. For example, Clp1’s localizations and functions at CRs and kinetochores were completely abrogated, while its mitotic control function was impaired and its CR protective function was preserved.

We found that both Clp1-Q111L, W113R and Clp1(1-372)-Q111L, W113R retain Clp1’s function in stabilizing the CR against perturbation. This role involves maintaining the septation initiation network (SIN) in an active state during late anaphase and requires Clp1 cytoplasmic localization and catalytic activity, but not Clp1 CR localization (Mishra *et al.*, 2004; Trautmann and McCollum, 2005). Because the Clp1 mutants localize to SPBs when the SIN is active, but not the CR, we surmise that the relevant substrate(s) for this Clp1 function resides on SPBs. Determining the identity and binding parameters of this substrate(s) may define Cdc14 substrate recognition mechanisms independent of the hydrophobic pocket. Indeed, the failure of the Clp1 mutants to persist at SPBs throughout interphase, unlike wild-type Clp1, has revealed at least two distinct SPB tethering mechanisms: one that is disrupted by the mutations we made in the hydrophobic pocket and another that is not.

Analysis of the Cdc25-4A mutant, which disrupts Clp1 binding and increases Cdc25 phosphorylation and abundance, affirms previous evidence (Esteban *et al.*, 2004; Wolfe and Gould, 2004) that Cdc25 is a direct Clp1 substrate. The reduced cell length of *cdc25-4A* cells further demonstrates that Clp1 regulates mitotic entry, at least in part, by modulating Cdk1 tyrosine phosphorylation via Cdc25 inhibition. This aligns with earlier work showing that Clp1 overproduction delays Cdk1 tyrosine dephosphorylation and mitotic entry (Trautmann *et al.*, 2001) and the identification of Cdc25 as a Clp1 substrate in a phosphoproteomic screen (Zeisner *et al.*, 2025). The intermediate mitotic control phenotypes of both the *cdc25-4A* and *clp1-Q111L, W113R* mutants relative to *clp1∆* likely reflect additional Clp1-dependent inhibition of Cdk1 via Wee1, affecting a parallel arm of the Cdk1 activation loop (Trautmann *et al.*, 2001), as well as Clp1 regulation of mitotic entry that is partially independent of the Cdk1 activation loop, as recently proposed (Zeisner *et al.*, 2025). The mechanistic basis for why Cdc25-4A is neither as phosphorylated nor as abundant as wild-type Cdc25 in *clp1∆* cells, however, remains less clear. Loss of Clp1 may alter the activity of other Cdc25 phosphatases, consistent with evidence for a temporally ordered cascade of phosphatase activation during mitosis (Grallert *et al.*, 2015; Touati *et al.*, 2019; Zeisner *et al.*, 2025). Still, clarifying how Clp1 integrates into this scheme will require further investigation.

Our study has revealed a major role for the C-terminus in Clp1 nucleolar localization, a result congruent with previous work showing that stress signaling promotes C-terminal phosphorylation-dependent release of Clp1 from the nucleolus (Diaz-Cuervo and Bueno, 2008; Broadus and Gould, 2012). However, because neither C-terminal truncation nor disruption of the hydrophobic pocket alone markedly affects nucleolar localization, whereas the combined perturbations do, we surmise that there is interplay between these Clp1 features that deserves further study. While nucleolar localization of *S. cerevisiae* Cdc14 might rely primarily on hydrophobic pocket-mediated association with Net1 (Kataria *et al.*, 2018), it has also been reported to depend on catalytic activity (Tomson *et al.*, 2009). Indeed, a multimodal mechanism of nucleolar targeting, as shown here for Clp1, may also be in play in multicellular eukaryotes because the N-terminal extension of human Cdc14B provides a nucleolar targeting cue (Mailand *et al.*, 2002).

In sum, our study has demonstrated a conserved role for the hydrophobic pocket of Cdc14 phosphatases and also revealed the existence of additional uncharacterized mechanisms by which Cdc14 phosphatases achieve their intracellular distribution and perform their full range of functions. Defining these previously unrecognized regulatory modes will be a fruitful area of future investigation and essential for understanding the diversification of Cdc14 phosphatase function across eukaryotes.

## MATERIALS AND METHODS

### Molecular biology methods

All cloning was performed by Gibson Assembly. *clp1, nsk1, mid1,* and *cdc25* sequences were amplified from genomic DNA or pre-existing plasmids and cloned into the BamHI and EcoRI sites of pMAL-c2 (New England Biolabs), pGEX2T (Cytiva), pGBT9, or pGAD424. In some cases, stop codons were introduced to produce shorter proteins. The open reading frame (ORF) of *nsk1*^+^ with 300 bp of 5′ and 3′ flanking sequences was cloned into the BamHI and SphI sites of pIRT2 (Booher and Beach, 1986). All point mutations, including those in pIRT2-*nsk1*, pIRT2-*mid1* (Clifford *et al.*, 2008b), pIRT2-*cdc25* (Lu *et al.*, 2012), and pIRT2-*clp1* (Wolfe *et al.*, 2006), were generated by site-directed mutagenesis using Pfu Turbo DNA polymerase (Catalogue # 600252, Agilent). PCR products and plasmids were sequenced by Plasmidsaurus using Oxford Nanopore Technology with custom analysis and annotation.

### Yeast methods

*S. pombe* strains used in this study were grown in yeast extract (YE) or Edinburgh minimal media (EMM) plus selective supplements (Forsburg and Rhind, 2006). Strain construction was accomplished through tetrad analysis using standard methods (Moreno *et al.*, 1991). Nuclei were visualized using 2 μg/ml 4′,6-diamidino-2-phenylindole (DAPI) with antifade on mounted cells after fixing cells in 70% ethanol overnight, followed by spinning down, aspirating, and resuspending in a 1 mg/ml stock of methyl blue (MB; Catalogue # M6900, Sigma). Expression of constructs under control of the thiamine-repressible *nmt* promoter system was regulated as previously described (Maundrell, 1993). Briefly, expression was repressed by the inclusion of 5 µM thiamine in agar plates or liquid media, and de-repression was achieved by washing cells in media lacking thiamine three times or plating on agar plates lacking thiamine.

Tagged strains were generated endogenously by adding the *mNG:kanMX6, mCherry:hphMX6, mCherry:natMX6, Myc_13_:kanMX6*, or *GFP:kanMX6* sequences internally or at the end of protein coding regions as previously described (Bahler *et al.*, 1998) using lithium acetate transformations (Keeney and Boeke, 1994). G418 (150 µg/ml; catalogue # 11811031, Sigma-Aldrich), hygromycin B (125 µg/ml, catalogue # 10687010, Life Technologies), or nourseothricin (clonNAT, 125 µg/ml, catalogue # N-500-100, GoldBio) was used for selection of *kanMX6, hphMX*6 cells, and *natMX6* cells, respectively. Tagged strains were confirmed by whole-cell PCR, live-cell imaging, and/or immunoblotting. All fusion proteins were expressed from their native promoters at their chromosomal loci unless otherwise noted.

*nsk1*, *clp1*, *mid1, and cdc25* mutant strains were generated by transforming *nsk1::ura4^+^*, *clp1::ura4^+^*, *mid1::ura4^+^*, or *cdc25*::*ura4^+^*/*cdc25^+^* cells, respectively, with pIRT2 plasmids or PCR fragments containing the desired mutations within the ORFs and 300–500 bp of sequences flanking the ORFs. pIRT2 transformants were selected on MA (minimal medium supplemented with adenine) agar plates, picked, and grown overnight in YE medium at 32°C. Approximately 1 OD_595_ of cells was plated on YE agar containing 1.5 g/l 5-fluoroorotic acid (5-FOA; catalogue # F5050, Toronto Research Chemicals) to select for loss of the *ura4* marker. Colonies growing on YE+5-FOA were streaked onto YE, MAU, and MAL plates to identify strains that had lost the pIRT2 plasmid and were uracil auxotrophs. Successful integrants were verified by whole-cell PCR using one primer located upstream or downstream of the homologous region and another within the internal ORF. PCR products were transformed via the lithium acetate method (Keeney and Boeke, 1994; Gietz *et al.*, 1995) and plated on YE agar containing 1.5 g/l 5-FOA to select for loss of the *ura4* marker. Correct integration events were verified by PCR and DNA sequencing.

Yeast two-hybrid analyses were performed using *S. cerevisiae* strain PJ69-4A and the pGBT9 and pGAD424 vectors (Clontech) as described (James *et al.*, 1996). Transformation of bait and prey plasmids containing various portions of *clp1^+^* or *nsk1*^+^ amplified by PCR from genomic DNA was performed by lithium acetate transformation (Gietz *et al.*, 1995). After transformation, cells were plated on SDMUHA (Synthetic Defined media supplemented with Methionine, Uracil, Histidine, and Adenine) agar plate to select cells containing both prey and bait plasmids. Leu^+^ and Trp^+^ transformants were then scored for positive interactions by streaking onto synthetic dextrose plates lacking histidine and synthetic dextrose plates lacking adenine and histidine. ß-galactosidase reporter enzyme activity in the transformants was also measured using the Galacto-Star chemiluminescent reporter gene assay system per manufacturer's instructions (Catalogue # BY100S, Applied Biosystems), except that cells were lysed by glass bead disruption using FastPrep-24 Sample Preparation System (Catalogue # 116004500, MP Biomedicals). Each sample was measured in triplicate. Reporter assays were recorded on a Synergy HT Plate Reader (Bio-TEK Instruments).

For growth assays, 10-fold serial dilutions were made in sterile PBS starting at 4.0 × 10^6^ cells/ml. 2.5 μl of each dilution was spotted on YE agar plates in the absence or presence of thiabendazole (Catalogue # T5535, Sigma) or latrunculin A (Catalogue # 10-2254, Focus Biomolecules).

### Protein expression and purification

Rosetta2(DE3)pLysS bacteria cells were grown in Terrific broth (23.6 g/l yeast extract, 11.8 g/l tryptone, 9.4 g/l K_2_HPO_4_, 2.2 g/l KH_2_PO_4_, 4 ml/l glycerol) supplemented with appropriate antibiotics to log-phase (OD_595_ 1–1.5) at 36°C. Cells were incubated on ice for 15 min, and protein expression was initiated with the addition of 0.4 mM isopropyl β-D-1-thiogalactopyranoside (IPTG; catalogue # BP1755, Fisher Scientific). Cells were then incubated for 16–18 h at 18°C for optimal protein production. Cells were harvested by centrifugation, snap frozen, and the cell pellets were kept at −80°C. Frozen cell pellets were lysed in either MBP buffer (20 mM Tris-HCl pH 7.4, 150 mM NaCl, 1 mM EDTA, protease inhibitor tablets [Catalogue # 05056489001, Roche]) or GST buffer (4.3 mM NaHPO_4_, 137 mM NaCl, 2.7 mM KCl, 1 mM dithiothreitol, pH 7.3, with the addition of cOmplete EDTA-free protease inhibitor cocktail and 0.1% NP-40 [catalogue # N3500, US Biologicals]). GST and MBP fusion proteins were purified with GST Bind Resin (Catalogue # 70541, EMD Millipore) and Amylose resin (Catalogue # E8021L, New England Biolabs), respectively, per manufacturers’ instructions.

### In vitro binding assays

In total, 5–15 μg recombinant GST or GST-fusion proteins conjugated to GST-bind resin were incubated in GST buffer supplemented with 5% BSA for 1 h, then incubated with 5 μg recombinant MBP or MBP-fusion proteins in 0.5 ml GST buffer for 1 h at 4°C. The beads were then washed with 1 ml GST buffer six times. When MBP or MBP-fusion proteins were on beads, the in vitro binding assays and washes were performed in MBP buffer instead. Bead-bound proteins were resuspended in 20 µl 2x SDS sample buffer, heated at 95°C for 5 min, and separated on a 4%–12% NuPAGE Bis-Tris gel with MOPS buffer (Catalogue # NP0322BOX, Invitrogen). Proteins were visualized by Coomassie blue staining and imaged on an Odyssey CLx instrument (LI-COR Biosciences).

### Cell lysate preparation, immunoprecipitation and immunoblotting

Yeast cell pellets (15 or 30 OD) were lysed by bead disruption using a FastPrep-24 cell homogenizer for 37s (4.0 m/s). For Cdc25-Myc_13_ and Nsk1-GFP immunoprecipitations (IPs), denatured lysates were made by adding 300 μl (500 μl for Nsk1-GFP pellets) SDS lysis buffer (6 mM Na_2_HPO_4_, 4 mM NaH_2_PO_4_, 0.5% SDS, 1 mM EDTA, 50 mM NaF, 1 mM DTT, 4 μg/ml leupeptin, 0.1 mM Na_3_VO_4_). For Nsk1-GFP IPs, Roche phosphatase inhibitor cocktail PhosStop and Roche protease inhibitor cocktail were also included. The lysates were then heated at 95°C for 90 s (120s for Nsk1-GFP lysates), cooled on ice, and then extracted by adding 800 μl NP-40 lysis buffer (6 mM Na_2_HPO_4_, 4 mM NaH_2_PO_4_, 1% NP-40, 150 mM NaCl, 50 mM NaF, 4 μg/ml leupeptin, 0.1 mM Na_3_VO_4_; Gould *et al.*, 1991) with the addition of 1 mM PMSF, 1.3 mM benzamidine, 0.2 mM diisopropyl fluorophosphate (DIFP), and Roche phosphatase and protease inhibitor cocktail. For Nsk1-GFP IPs, two extractions with 500 μl NP-40 lysis buffer were done. Lysates were cleared by centrifugation at 2000 × *g* for 5 min (9600 × *g* for 6 min for Nsk1-GFP lysates). For immunoprecipitations, 4 μg of mouse monoclonal anti-Myc antibody 9E10 or 2 μg of rabbit polyclonal anti-GFP antibody was added to cleared cell lysates and incubated for 1 h at 4°C, followed by adding 80 μl 50% slurry of protein G sepharose beads (Catalogue # 17061802, GE Healthcare) for Cdc25-Myc_13_ or 50 uL 50% slurry of nProtein A sepharose beads (Catalogue # 17528004, GE Healthcare) for Nsk1-GFP and incubation for another 1 h. The beads were washed three times with 1 ml NP-40 buffer (only twice for Nsk1-GFP), and proteins were eluted with SDS sample buffer. In case of λ-phosphatase treatment, the beads were washed twice with 1 ml of 1X PMP buffer (50 mM HEPES, 100 mM NaCl, 2 mM DTT, 0.01% Brij 35, pH 7.5), followed by incubation with 1 μl λ-phosphatase (Catalogue # P0753S, NEB) in 1X PMP buffer containing 1 mM MnCl_2_ (total volume 20 μl) at 30°C for 45 min with shaking. For Clp1-GFP, 15 OD of cell pellets were lysed with FastPrep-24 cell homogenizer, and straight cell lysates were made by extraction with 800 μl urea lysis buffer (8 M urea, 50 mM Tris pH 8.5, 1 mM PMSF, 1.3 mM benzamidine, and protease inhibitor cocktail tablet). Lysates were cleared by centrifugation at 13,000 rpm for 5 min. Proteins were separated on NuPAGE 4-12% Bis-Tris gels (Catalogue # NP0321BOX, Invitrogen) with MOPS SDS running buffer (Catalogue # NP0001-02, Invitrogen; 8% SDS–PAGE gel for Nsk1-GFP), transferred to Immobilon-P PVDF membranes (Catalogue # IPVH00005, Millipore Sigma) with NuPAGE transfer buffer (Catalogue # NP00061, Invitrogen; 30V for 1.5 h) or iBlot system (Invitrogen, program P0 for 7 mins), and immunoblotted with 9E10 anti-myc (monoclonal, Vanderbilt Antibody and Protein Resource Core; 0.5 μg/ml), anti-GFP (Proteintech catalogue # 50430-2-AP, RRID:AB_11042881; 0.5–1 μg/ml), anti-α-tubulin (Sigma-Aldrich catalogue # T5168, RRID:AB_477579; 1:10,000), or anti-Cdc2 antibody PSTAIRE (1:10,000), followed by anti-mouse or anti-rabbit secondary antibodies conjugated to IRDye 680 or IRDye 800 (LI-COR Biosciences) (1:20,000) and imaged with an Odyssey CLx instrument (LI-COR Biosciences). For Nsk1-GFP IPs, after transferring, the membrane was air-dried for at least 1 h before proceeding with immunoblotting.

### Microscopy

Images were acquired using 1) a Personal DeltaVision microscope system (Leica Microsystems), which includes an Olympus IX71 microscope, a 60 × 1.42 NA PlanApo oil immersion objective, a 60 × 1.49 TIRF objective, a pco.edge sCMOS camera, and softWoRx imaging software, 2) a Zeiss Axio Observer inverted epifluorescence microscope with a Zeiss 63X oil (1.46 NA) objective, Zeiss ZEN 3.0 (Blue edition) software and an Axiocam 503 monochrome camera (Zeiss), or 3) a Leica Thunder imager system including a DMI8 inverted microscope, 63X plan apo oil objective (1.40 NA), a Leica K8 sCMOS camera, standard excitation and emission filters, and an LED light source (Leica Microsystems). Images were acquired using Leica Application Suite X (LAS X) software (Leica Microsystems). Exposure times ranged from 100 to 250 ms. Z-slices were acquired with 0.5 μm spacing for a total distance of 4.5–5.5 μm. Images acquired on the DeltaVision were deconvolved with 10 iterations unless otherwise noted. Images for quantification of fluorescence intensity were not deconvolved and were sum-projected. Intensity measurements were made with Fiji (a version of ImageJ software available at https://fiji.sc; Schindelin *et al.*, 2012), with each image corrected for camera background. A region of interest was created to measure the intensity within the cell. Cell lengths were measured from single medial slices using Fiji. Line segments between each pole of the cell along the midpoint of the diameter were drawn to determine cell lengths at septation. Line scans were performed on a single medial z-slice of images without deconvolution in ImageJ. A 5 µm line was drawn across the area of interest, and the fluorescence intensities of each channel along the line were obtained and exported to GraphPad Prism for final plotting.

Fluorescence recovery after photobleaching (FRAP) analysis was performed on the Leica Thunder microscope described above. A single medial z-slice was acquired every 1s for 30s, and three images were taken before the photobleaching event. A 0.35 µm^2^ spot of nucleolar Clp1-GFP or a region encompassing the entire nucleolus was photobleached with 20 interactions using 10% power of a 488 laser. Quantification of the fluorescence recovery was performed using Fiji. FRAP intensity measurements were corrected for background and time-course photobleaching. The halftime (T_1/2_), mobile fraction (M_f_), and statistics were calculated using Microsoft Excel or Prism 8.0 software (Graphpad software).

### Protein structure prediction and sequence alignment

Protein structure predictions were generated with the AlphaFold3 server (https://alphafoldserver.com) (Abramson *et al.*, 2024) using protein sequences from Pombase (Carme *et al.*, 2026). The automatic seed setting was used for all predictions. For protein-protein interaction analysis, the top-ranked “model_0″ prediction was selected to extract interface information and visualization using the PyMOL molecular graphics system (version 3.0, Schrödinger). Protein sequence alignments were performed using Clustal Omega (Sievers and Higgins, 2021).

### Statistical analyses

Calculations of mean, standard error of the mean (SEM), SD (SD), and statistical significance were performed with Prism 8.0 (GraphPad Software). Significance was defined by a *p* value equal to or less than 0.05. One-way ANOVA was used with Tukey's post hoc test for multiple comparisons. No data were excluded from the analysis. Unless indicated differently in the figure legends, all experiments were performed twice, and one representative result is shown.

## DATA AVAILABILITY STATEMENT

The data underlying Figures and Supplemental Figures are openly available in Mendeley Data at doi: 10.17632/9rw2nb7swt.1; DOI: 10.17632/nxg3m8ddcs.1; DOI: 10.17632/435mkknh97.1.

## Supporting information





## Abbreviations used

AF: AlphaFold3
Cdk1: cyclin-dependent kinase 1
CR: cytokinetic ring
DIC: differential interference contrast
FRAP: fluorescence recovery after photobleaching
MB: methyl blue
SIN: septation initiation network
SLiM: short linear interaction motif
SPB: spindle pole body
YE: yeast extract.
