# Supplementary material for: Conserved and divergent modes of substrate interaction define selective localizations and functions of a Cdc14 phosphatase
Source: Mol Biol Cell. 2026 Sep 25;37(10):ar95. doi: 10.1091/mbc.E26-04-0156 (PMC13637742; doi:10.1091/mbc.E26-04-0156)
Supplement: Supplementary file 1 [file mbc-37-ar95-s001.pdf]

Supplemental Materials

Molecular Biology of the Cell

Chen *et al*.

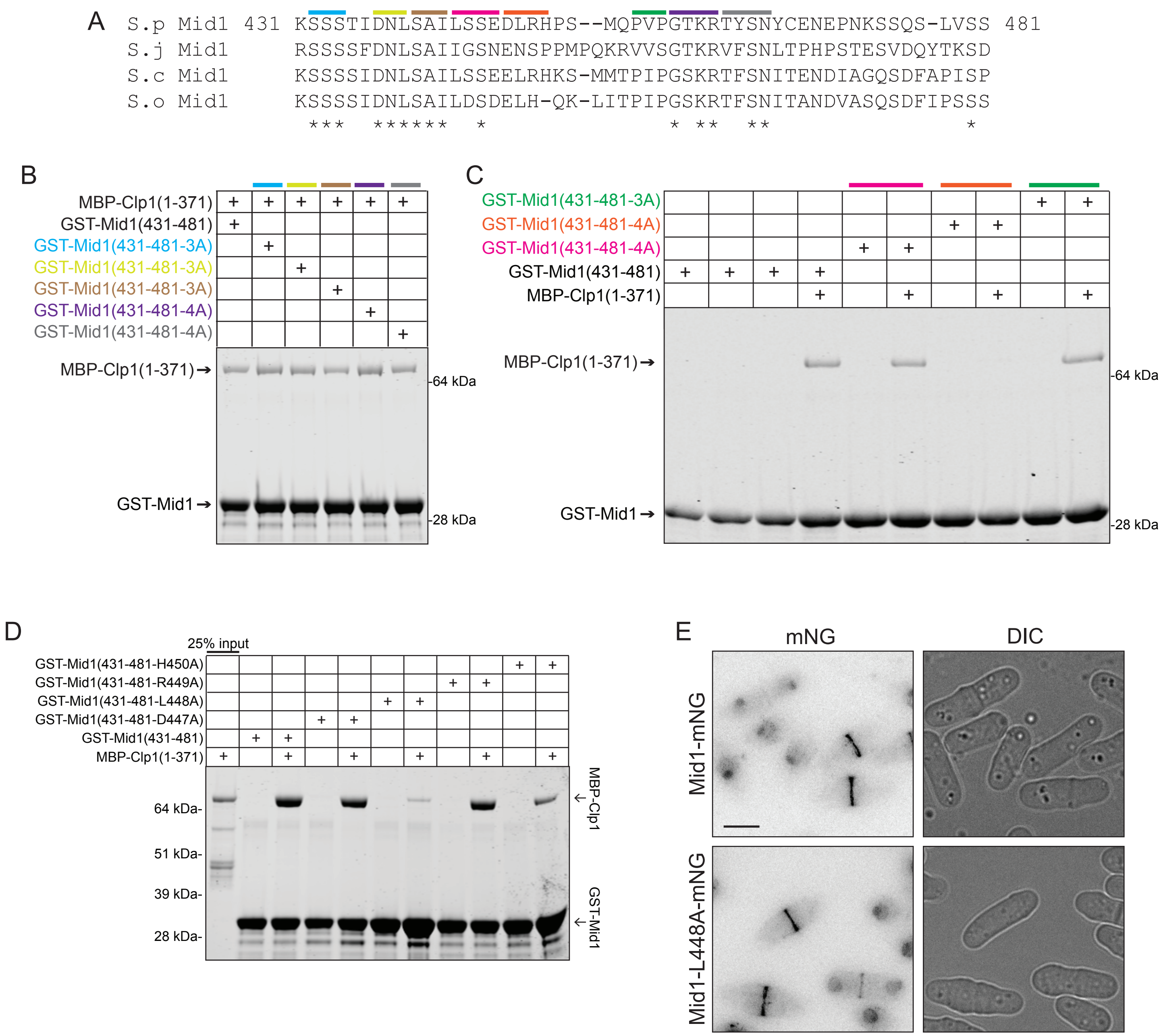
A
S.p Mid1 431 KSSSTIDNLSAILSSEDLRHPS--MQPVPGTKRTYSNYCENEPNKSSQS-LVSS 481
S.j Mid1 RSSSSFDNLSAIIGSNENSPPMPQKRVVSGTKRVFSNLTPHPSTESVDQYTKSD
S.c Mid1 KSSSSIDNLSAILSSEELRHKS-MMTPIPGSKRTFSNITENDIAGQSDFAPISP
S.o Mid1 KSSSSIDNLSAILDSDELH-QK-LITPIPGSKRTFSNITANDVASQSDFIPSSS
B
MBP-Clp1(1-371)
GST-Mid1(431-481)
GST-Mid1(431-481-3A)
GST-Mid1(431-481-3A)
GST-Mid1(431-481-3A)
GST-Mid1(431-481-4A)
GST-Mid1(431-481-4A)
MBP-Clp1(1-371)→
GST-Mid1→
-64 kDa
-28 kDa
C
GST-Mid1(431-481-3A)
GST-Mid1(431-481-4A)
GST-Mid1(431-481-4A)
GST-Mid1(431-481)
MBP-Clp1(1-371)
MBP-Clp1(1-371)→
GST-Mid1→
-64 kDa
-28 kDa
D
25% input
GST-Mid1(431-481-H450A)
GST-Mid1(431-481-R449A)
GST-Mid1(431-481-L448A)
GST-Mid1(431-481-D447A)
GST-Mid1(431-481)
MBP-Clp1(1-371)
64 kDa-
51 kDa-
39 kDa-
28 kDa-
MBP-Clp1
GST-Mid1
E
mNG
DIC
Mid1-mNG
Mid1-L448A-mNG

Figure S1

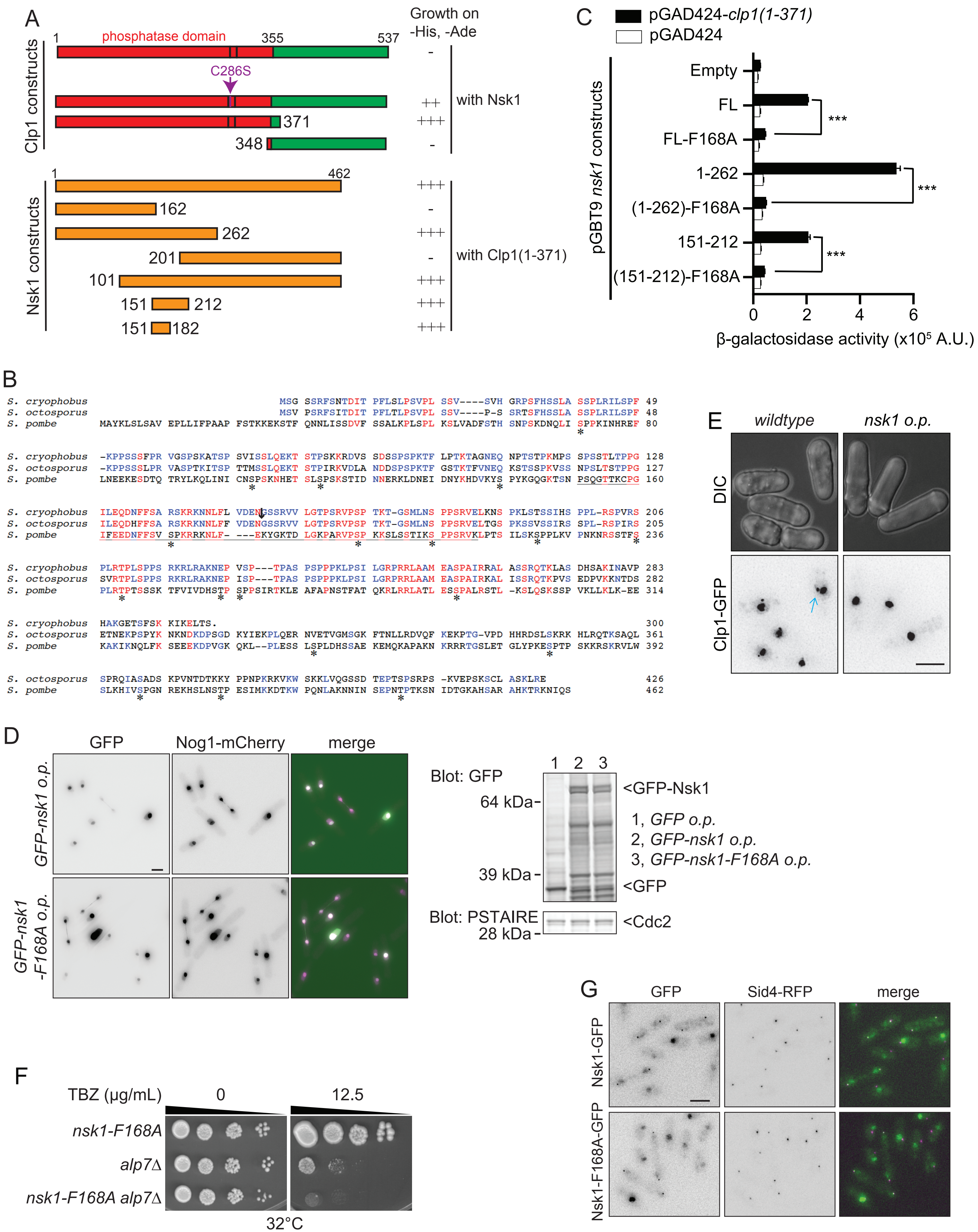
A
Growth on -His, -Ade
Clp1 constructs
1
phosphatase domain
355
537
C286S
371
348
-
++
+++
-
with Nsk1
Nsk1 constructs
1
462
162
262
201
101
151
212
182
+++
-
+++
-
+++
+++
+++
with Clp1(1-371)
C
pGAD424-clp1(1-371)
pGAD424
pGBT9 nsk1 constructs
Empty
FL
FL-F168A
1-262
(1-262)-F168A
151-212
(151-212)-F168A
***
0
2
4
6
β-galactosidase activity (x10⁵ A.U.)
B
S. cryophobus MSG SSRFSNTDIT PFLSLPSVPL SSV----SVH GRPSFHSSLA SSPLRILSPF 49
S. octosporus MSV PSRFSITDIT PFLTLPSVPL SSV----P-S SRTSFHSSLA SSPLRILSPF 48
S. pombe MAYKLSLSAV EPLLIFPAAP FSTKKEKSTF QNNLISSDVF SSALKPLSPL KSLVADFSTH SNPSKDNQLI SPPKINHREF 80
S. cryophobus -KPPSSSFPR VGSPSKATSP SVISSLQEKT STPSKKRDVS SDSSPSPKTF LPTKTAGNEQ NPTSTPKMPS SPSSTLTPPG 128
S. octosporus -KPPSSSLPR VASPTKITSP TTMSSLQEKT STPIRKVDLA NDDSPSPKTF GSTKTFVNEQ KSTSSPKVSS NPSLTSTPPG 127
S. pombe LNEEKESDTQ TRYLKQLINI CNSPSKNHET SLSPSKSTID NNERKLDNEI DNYKHDVKYS PYKGQGKTSN PSQGTTKCPG 160
S. cryophobus ILEQDNFFSA RSKRKNNLFL VDENGSSRVV LGTPSRVPSP TKT-GSMLNS PPSRVELKNS PKLSTSSIHS PPL-RSPVRS 206
S. octosporus ILEQDHFFSA RSKRKNNLFF VDENGSSRVV LGTPSRVPSP TKT-GSMLNS PPSRVELTGS PKSSVSSIRS SPL-RSPIRS 205
S. pombe IFEEDNFFSV SPKRRKNLF- ---EKYGKTD LGKPARVPSP KKSLSSTIKS PPSRVKLPTS ILSKSPPLKV PNKNRSSTFS 236
S. cryophobus PLRTPLSPPS RKRLRAKNEP VSP---TPAS PSPPPKLPSI LGRPRRLAAM EASPAIRRAL ASSRQTKLAS DHSAKINAVP 283
S. octosporus SVRTPLSPPS RKRLRAKNEP ISP---TPAS PSPPPKLPSI LGRPRRLAAM EASPAIRKAL ISSRQTKPVS EDPVKKNTDS 282
S. pombe PLRTPTSSSK TFVIVDHSTP SPPSIRTKLE AFAPNSTFAT QKRLRRLATL ESSPALRSTL -KSLQSKSSP VKLLKLK-EE 314
S. cryophobus HAKGETSFSK KIKELTS. 300
S. octosporus ETNEKPSPYK NKNDKDPSGD KYIEKPLQER NVETVGMSGK FTNLLRDVQF KEKPTG-VPD HHRDSLSKRK HLRQTKSAQL 361
S. pombe KAKIKNQLFK SEEEKDPVGK QKL--PLESS LSPLDHSSAE KEMQKAPAKN KRRRTGSLET GLYPKESPTP SKKRSKRVLW 392
S. octosporus SPRQIASADS KPVNTDTKKY PPNPKRKVKW SKKLVQGSSD TEPTSPSRPS -KVEPSKSCL ASKLRE 426
S. pombe SLKHIVSPGN REKHSLNSTP ESIMKKDTKW PQNLAKNNIN SEPNTPTKSN IDTGKAHSAR AHKTRKNIQS 462
E
wildtype
nsk1 o.p.
DIC
Clp1-GFP
D
GFP
Nog1-mCherry
merge
GFP-nsk1 o.p.
GFP-nsk1 -F168A o.p.
Blot: GFP
1 2 3
64 kDa
39 kDa
<GFP-Nsk1
1, GFP o.p.
2, GFP-nsk1 o.p.
3, GFP-nsk1-F168A o.p.
<GFP
Blot: PSTAIRE
28 kDa
<Cdc2
F
TBZ (μg/mL)
0
12.5
nsk1-F168A
alp7Δ
nsk1-F168A alp7Δ
32°C
G
GFP
Sid4-RFP
merge
Nsk1-GFP
Nsk1-F168A-GFP

Figure S2

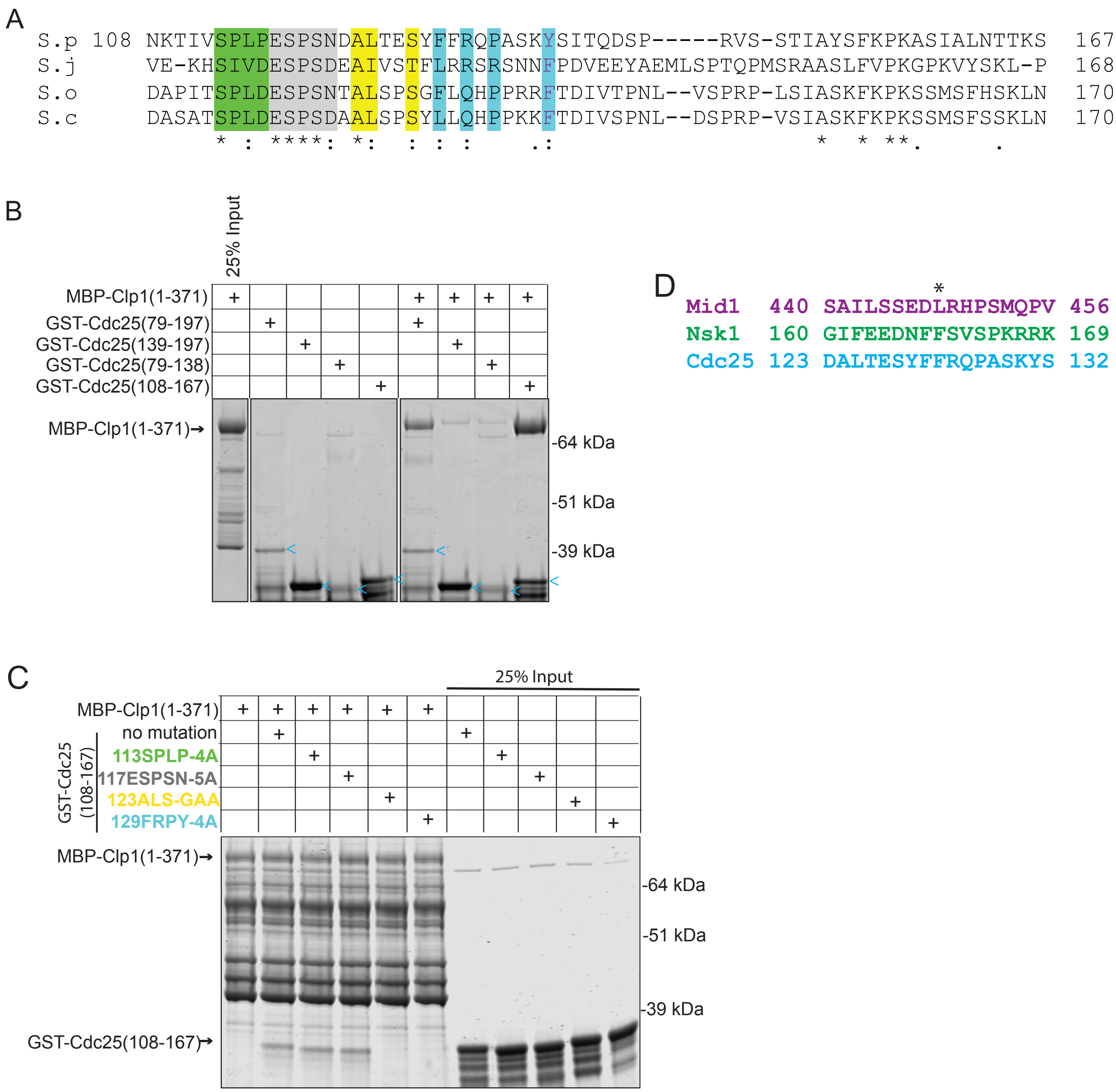
A
S.p 108 NKTIVSPLPESPSNDALTESYFFRQPASKYSITQDSP-----RVS-STIAYSFKPKASIALNTTKS 167
S.j VE-KHSIVDESPSDEAIVSTFLRRSRSNNFPDVEEYAEMLSPTQPMSRAASLFVPKGPKVYSKL-P 168
S.o DAPITSPLDESPSNTALSPSGFLQHPPRRFTDIVTPNL--VSPRP-LSIASKFKPKSSMSFHSKLN 170
S.c DASATSPLDESPSDAALSPSYLLQHPPKKFTDIVSPNL--DSPRP-VSIASKFKPKSSMSFSSKLN 170
B
25% Input
MBP-Clp1(1-371)
GST-Cdc25(79-197)
GST-Cdc25(139-197)
GST-Cdc25(79-138)
GST-Cdc25(108-167)
MBP-Clp1(1-371)→
-64 kDa
-51 kDa
-39 kDa
C
25% Input
MBP-Clp1(1-371)
no mutation
GST-Cdc25 (108-167)
113SPLP-4A
117ESPSN-5A
123ALS-GAA
129FRPY-4A
MBP-Clp1(1-371)→
GST-Cdc25(108-167)→
-64 kDa
-51 kDa
-39 kDa
D
Mid1 440 SAILSSEDLRHPSMQPV 456
Nsk1 160 GIFEEDNFFSVSPKRRK 169
Cdc25 123 DALTESYFFRQPASKYS 132

Figure S3

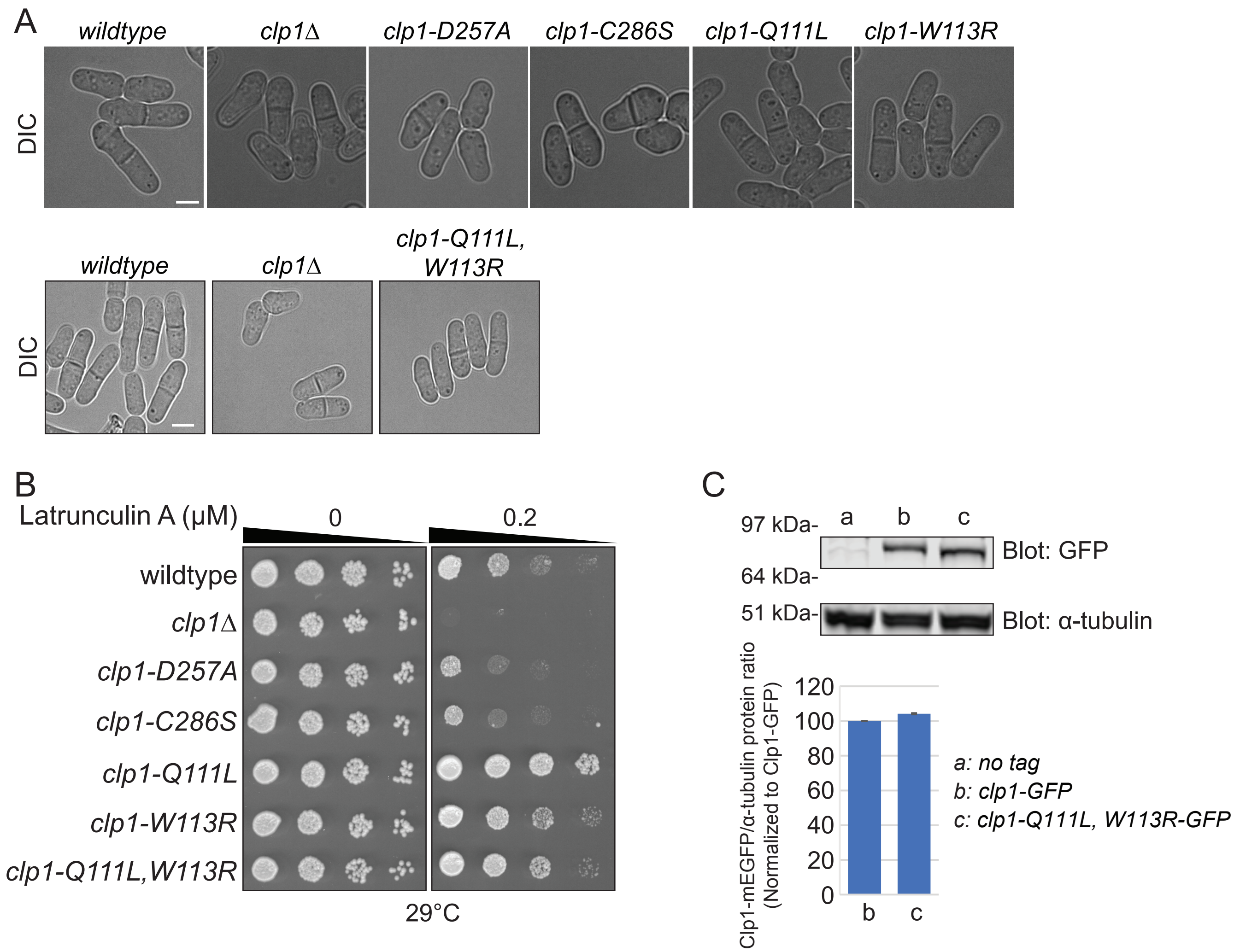
A
wildtype
clp1Δ
clp1-D257A
clp1-C286S
clp1-Q111L
clp1-W113R
DIC
wildtype
clp1Δ
clp1-Q111L, W113R
DIC
B
Latrunculin A (μM)
0
0.2
wildtype
clp1Δ
clp1-D257A
clp1-C286S
clp1-Q111L
clp1-W113R
clp1-Q111L,W113R
29°C
C
97 kDa-
64 kDa-
51 kDa-
a
b
c
Blot: GFP
Blot: α-tubulin
Clp1-mEGFP/α-tubulin protein ratio (Normalized to Clp1-GFP)
120
100
80
60
40
20
0
b
c
a: no tag
b: clp1-GFP
c: clp1-Q111L, W113R-GFP

Figure S4

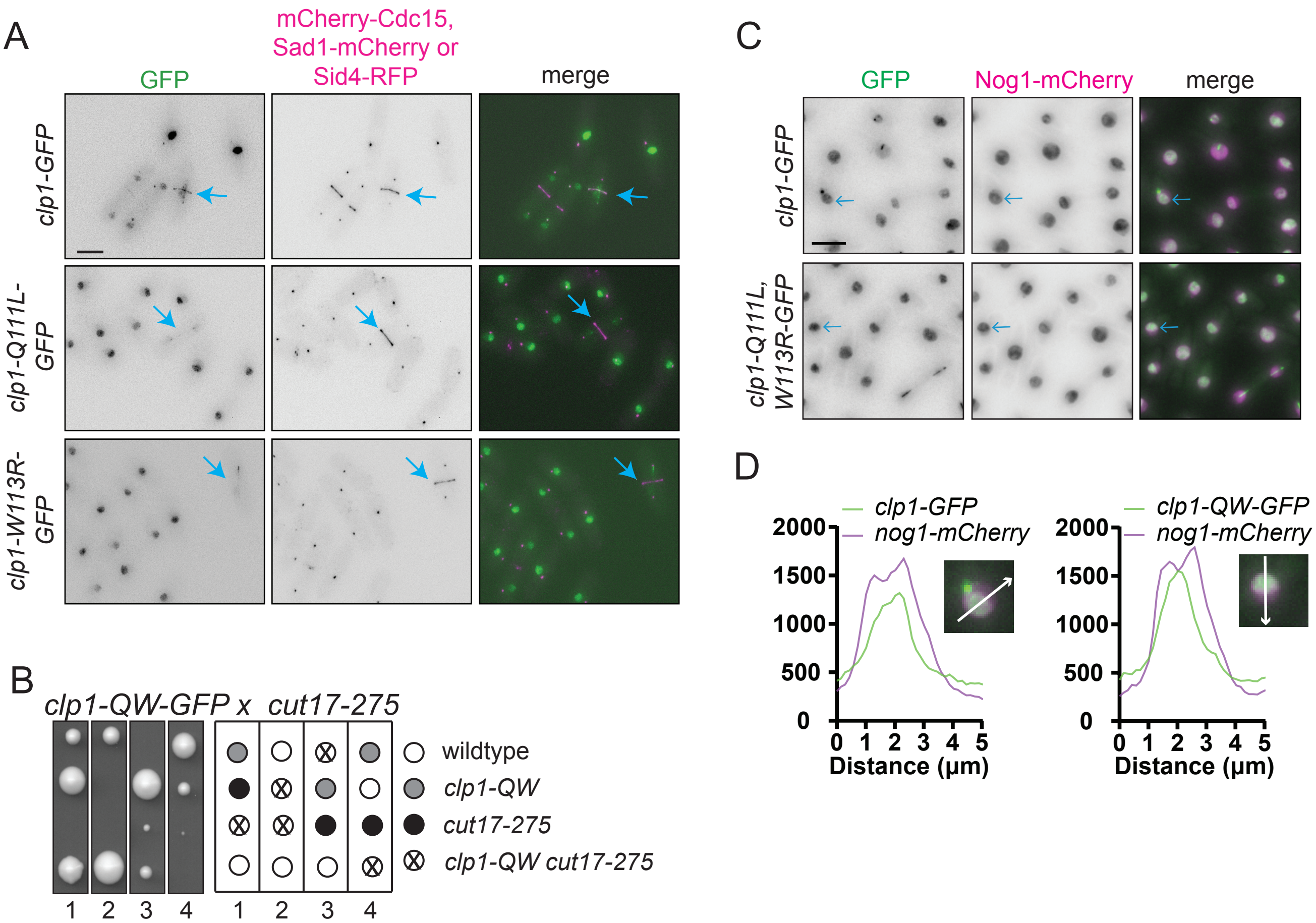
A
GFP
mCherry-Cdc15, Sad1-mCherry or Sid4-RFP
merge
clp1-GFP
clp1-Q111L-GFP
clp1-W113R-GFP
B
clp1-QW-GFP x cut17-275
wildtype
clp1-QW
cut17-275
clp1-QW cut17-275
1 2 3 4
1 2 3 4
C
GFP
Nog1-mCherry
merge
clp1-GFP
clp1-Q111L, W113R-GFP
D
clp1-GFP
nog1-mCherry
clp1-QW-GFP
nog1-mCherry
2000
1500
1000
500
0
0 1 2 3 4 5
Distance (μm)

Figure S5

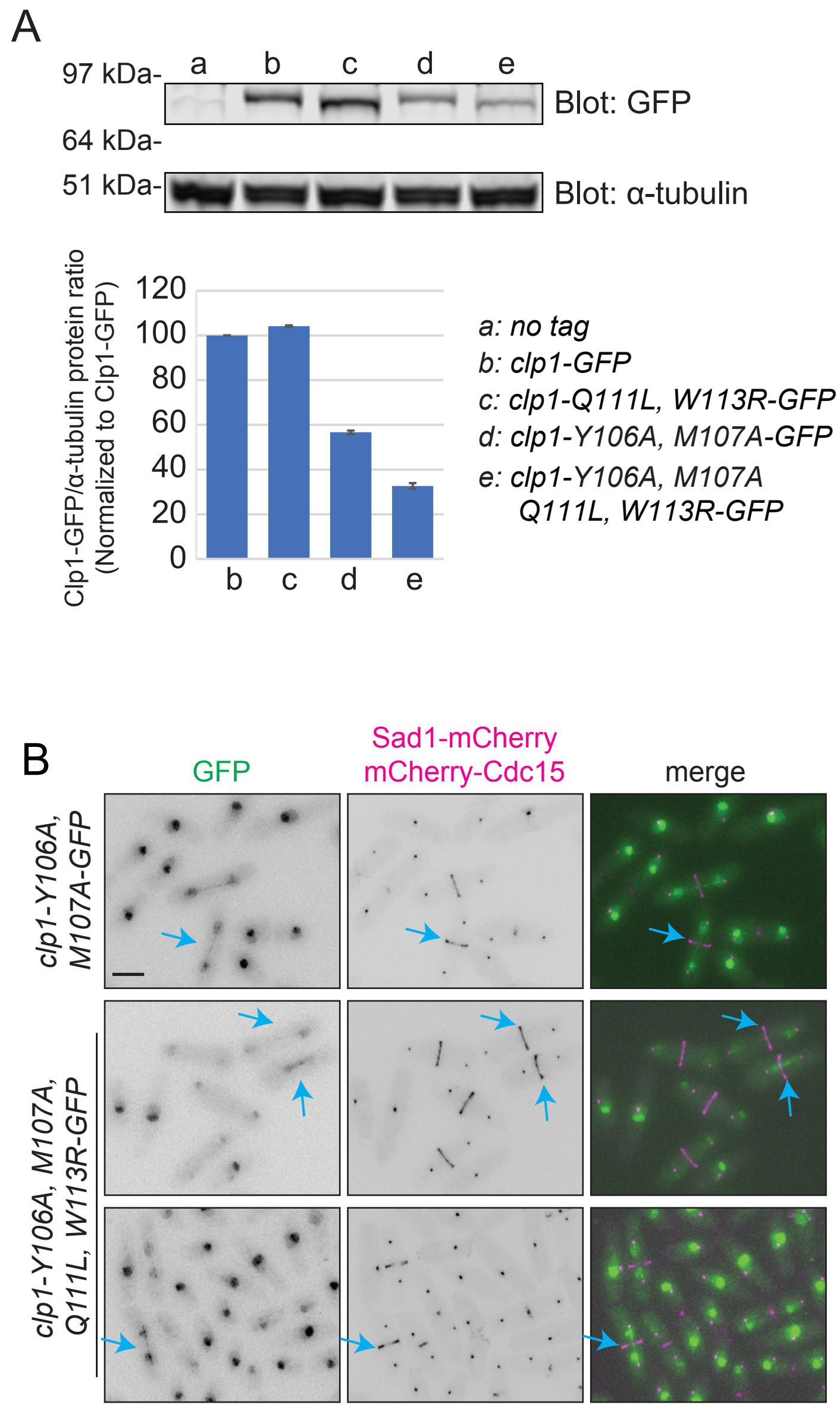
A
97 kDa-
a
b
c
d
e
Blot: GFP
64 kDa-
51 kDa-
Blot: α-tubulin
Clp1-GFP/α-tubulin protein ratio
(Normalized to Clp1-GFP)
120
100
80
60
40
20
0
b
c
d
e
a: no tag
b: clp1-GFP
c: clp1-Q111L, W113R-GFP
d: clp1-Y106A, M107A-GFP
e: clp1-Y106A, M107A
Q111L, W113R-GFP
B
GFP
Sad1-mCherry
mCherry-Cdc15
merge
clp1-Y106A, M107A-GFP
clp1-Y106A, M107A, Q111L, W113R-GFP

Figure S6

Figure S1. Definition of Mid1-Clp1 interaction region. A) Sequence alignment of four *Schizosaccharomyces* Mid1 protein fragments generated using Clustal Omega [1,2]. Asterisks indicate identical residues. The colored lines denote the different amino acid clusters mutated for in vitro binding experiments. *S. pombe* (S.p), *S. japonicus* (S.j), *S. cryophilus* (S.c) *and S. octosporus* (S.o). B-D) Bead-bound GST-Mid1(431-481) or variants thereof were incubated with buffer alone or with MBP-Clp1(1-371) for 1 hour prior to washing. Samples were then resolved by SDS-PAGE and gels were stained with Coomassie blue before imaging with an Odyssey CLx instrument. The color of the GST-Mid1 constructs in panels B and C matches the line color in A. E) Representative live-cell images of the indicated strains grown at 25°C. Scale bar, 5 μm.

Figure S2. Definition of Nsk1-Clp1 interaction regions. A) *S. cerevisiae* strain PJ69-4A was co-transformed with plasmids expressing the indicated fragments of *nsk1*, *clp1*, or their variants. The position of the Clp1 catalytic center is indicated with two black bars and the position of the substrate-trapping catalytically inactive C286S mutant is provided. Transformants were scored for growth on plates lacking histidine and adenine. Three pluses indicate strong growth, two pluses indicate moderate growth, and minuses indicate no growth. B) Sequence alignment of *S. pombe* Nsk1 with its homologs, with which it shares ~23% overall sequence identity. Identical and highly conserved residues amongst all three proteins are in red. Residues identical in two proteins are in blue. Nsk1 contains two putative coiled-coils spanning amino acids 114-141 and 290-331. The identified Cdk1 phosphorylation sites (SP/TP) in Nsk1 [3] are indicated with asterisks, the Clp1 interacting region of 151-212 is underlined, and F168 is indicated with an arrow. C) The β-galatosidase activity of *S. cerevisiae* strain PJ69-4A transformants containing the indicated bait and prey plasmids was determined in triplicate (represented in arbitrary light units (A.U.)). Error bars represent SEM. *** $p < 0.001$ by Student's *t*-test. D) (left) Representative live-cell images of the indicated strains grown at 32˚C. Cells were initially grown in media containing thiamine and then washed into media without thiamine and grown for 24

hours prior to imaging. At the 24-hour timepoint, samples were also taken for immunoblot analysis of GFP proteins with α-PSTAIRE as loading control (right panels). E) Representative live-cell images of *clp1-GFP* cells grown at 32˚C with repressed or induced *nsk1⁺* expression for 24 h prior to imaging. The blue arrow points to an example of Clp1 localized at a SPB. DIC; Differential Interference Contrast. F) The indicated strains were grown to mid-log phase and spotted in 10-fold serial dilutions on YE plates containing 12.5 µg/mL thiabendazole (TBZ) or DMSO (solvent control). The plate was incubated at 32°C for 2-4 days. G) Representative live-cell images of the indicated strains. Scale bars, 5 µm.

Figure S3. Definition of Cdc25-Clp1 interaction region. A) Sequence alignment of *S. pombe* (S.p) Cdc25 residues 108-167 with Cdc15 proteins from *S. japonicus* (S.j), *S. cryophilus* (S.c) and *S. octosporus* (S.o) using Clustal Omega [1,2]. Asterisks indicate identical residues. Each group of residues targeted for mutagenesis is shaded in a distinct color. ":" indicates conservation of strongly similar residues and "." indicates conservation of weakly similar residues. B) Bead-bound GST-Cdc25 fragments were incubated with MBP-Clp1(1-371) for 1 hour prior to washing. Samples were then resolved by SDS-PAGE and gels were stained with Coomassie blue before imaging with an Odyssey CLx instrument. Blue arrowheads indicate the positions of the GST-Cdc25 fragments. C) Bead-bound MBP-Clp1(1-371) was incubated with GST-Mid1(431-481) or variants thereof for 1 h prior to washing. Samples were then resolved by SDS-PAGE and gels were stained with Coomassie blue before imaging with an Odyssey CLx instrument. D) Sequence alignment of the indicated regions of Mid1, Nsk1 and Cdc25 proteins generated by Clustal Omega [1,2]. The asterisk indicates the key residue in each protein for Clp1 binding.

Figure S4. Characterization of Clp1 mutants. A) Representative live-cell images of the indicated strains grown at 25°C in YE media. DIC; Differential Interference Contrast. B) The

indicated strains were grown to mid-log phase, spotted in 10-fold serial dilutions on YE plates containing 0.2 μM latrunculin A or DMSO (solvent control), and incubated at 29°C for 3 days. C) Representative immunoblot of Clp1-GFP proteins with α-tubulin as loading control (top panel). Quantification from two independent immunoblotting experiments normalized to Clp1-GFP (bottom panel). Error bars represent SD.

Figure S5. Characterization of Clp1 mutants. A and C) Representative live-cell images of the indicated strains. Scale bars, 5 μm. B) Four tetrads from the indicated cross incubated at 25˚C on YE plates and a schematic of relevant genotypes to the right. QW, Q111L,W113R. D) Line scan plots of the cells indicated by blue arrows from C. Zoomed views of the nuclei are shown, with the white arrows denoting the position and direction of the line scans performed. The white arrows also serve as scale bars equaling 5 μm.

Figure S6. Characterization of additional Clp1 mutants. A) Representative immunoblot of Clp1-GFP proteins with α-tubulin as loading control (top panel). A portion of this blot and its quantification was also presented in Figure S4C. Quantification from two independent immunoblotting experiments normalized to Clp1-GFP (bottom panel). Error bars represent SD. B) Representative live-cell images of the indicated strains. Blue arrows indicate the positions of fully formed CRs that have not yet begun constriction. Scale bar, 5 μm.

References

1. Harris, M.A., Rutherford, K.M., Hayles, J., Lock, A., Bahler, J., Oliver, S.G., Mata, J., and Wood, V. (2022). Fission stories: using PomBase to understand Schizosaccharomyces pombe biology. Genetics *220*. 10.1093/genetics/iyab222.
2. Sievers, F., and Higgins, D.G. (2021). The Clustal Omega Multiple Alignment Package. Methods Mol Biol *2231*, 3-16. 10.1007/978-1-0716-1036-7_1.
3. Chen, J.S., Broadus, M.R., McLean, J.R., Feoktistova, A., Ren, L., and Gould, K.L. (2013). Comprehensive proteomics analysis reveals new substrates and regulators of the fission yeast clp1/cdc14 phosphatase. Molecular & cellular proteomics : MCP *12*, 1074-1086. 10.1074/mcp.M112.025924.

TABLE S1 *S. pombe* strains used in this study

Strain #	Genotype	Source
**Figure 1**		
KGY33-2	*clp1-mEGFP:kanMX6 sad1-mCherry:natMX6 mCherry-cdc15 ade6-M21X leu1-32 ura4-D18* h^+	This study
KGY445-2	*clp1(1-372)-mEGFP:kanMX6 sad1-mCherry:natMX6 mCherry-cdc15 ade6-M21X leu1-32 ura4-D18* h^+	This study
KGY881-2	*clp1-EGFP:kanMX6 mCherry-cdc15 sid4-RFP:kanMX6 ade6-M21X leu1-32 ura4-D18* h^+	This study
KGY299-2	*mid1-L448A clp1-EGFP:kanMX6 mCherry-cdc15 sid4-RFP:kanMX6 ade6-M21X leu1-32 ura4-D18* h^+	This study
**Figure 2**		
KGY12755	*clp1-EGFP:kanMX6 cdc15-mCherry:kanMX6 sid4-RFP:kanMX6 ade6-M21X leu1-32 ura4-D18* h^+	This study
KGY11883	*clp1-EGFP:kanMX6 leu1+::P3nmt1-nsk1-T.nmt1:leu1-32 cdc15-mCherry:kanMX6 sid4-RFP:kanMX6 ade6-M210 leu1-32 ura4-D18* $h^?$	This study
KGY723-2	*clp1-EGFP:kanMX6 leu1+::P3nmt1-nsk1-F168A-T.nmt1:leu1-32 cdc15-mCherry:kanMX6 sid4-RFP:kanMX6 ade6-M21X leu1-32 ura4-D18* h^+	This study
KGY5991	*nsk1-EGFP:kanMX6 nda3-KM311* h^-	Lab stock
KGY9298-2	*nsk1-F168A-EGFP:kanMX6 nda3-KM311 leu1-32 ade6-M210? ura4-D18?* h^-	This study
**Figure 3**		
KGY246	*ura4-D18 ade6-M210 leu1-32* h^-	Lab stock
KGY223-2	*clp1Δ::kanMX6 ade6-M210 ura4-D18 leu1-32 h?*	Lab stock
KGY1904-2	*cdc25-4A (F129A,R131A,P133A,Y137A) ade6-M21X ura4-D18 leu1-32* h^+	This study
KGY3239-2	*cdc25-Myc*$_{13}$*:kanMX6 ura4-D18 ade6-M210 leu-32* h^-	This study
KGY3244-2	*cdc25-Myc*$_{13}$*:kanMX6 clp1Δ::ura4*$^+$ *ura4-D18 ade6-M216 leu-32* h^-	This study
KGY378-2	*cdc25-4A-Myc*$_{13}$*:kanMX6 (F129A,R131A,P133A,Y137A) ade6-M21X ura4-D18 leu1-32* h^+	This study
KGY881-2	*clp1-EGFP:kanMX6 mCherry-cdc15 sid4-RFP:kanMX6 ade6-M210 leu1-32 ura4-D18* h^+	This study
KGY33-2	*clp1-mEGFP:kanMX6 sad1-mCherry:natMX6 mCherry-cdc15 ade6-M21X leu1-32 ura4-D18* h^+	This study
**Figure 5**		
KGY246	*ura4-D18 ade6-M210 leu1-32* h^-	Lab stock
KGY223-2	*clp1Δ::kanMX6 ura4-D18 ade6-M210 leu1-32 h?*	Lab stock
KGY4587	*clp1-D257A ura4-D18 ade6-M210 leu1-32* h^-	Lab stock
KGY4972	*clp1-C286S ura4-D18 ade6-M210 leu1-32* h^-	Lab stock
KGY1-2	*clp1-Q111L ura4-D18 ade6-M210 leu1-32* h^+	This study
KGY19942	*clp1-W113R ura4-D18 ade6-M210 leu1-32* h^+	This study
KGY1421-2	*clp1-Q111L,W113R ade6-M21X ura4-D18 leu1-32* h^-	This study
KGY17141	*cps1-191 ura4-D18 leu1-32 ade6-M210* h^+	Lab stock
KGY4791-2	*cps1-191 clp1Δ::ura4+ ura4-D18 ade6-M21X leu1-32 lys1-151* h^-	Lab stock
KGY863-2	*cps1-191 clp1-D257A ade6-M21X ura4-D18 leu1-32* h^+	This study
KGY2038-2	*cps1-191 clp1-C286S-TAP:kanMX6 ade6-M210 ura4-D18 leu1-32* h^+	This study
KGY885-2	*cps1-191 clp1-Q111L ade6-M21X ura4-D18 leu1-32* h^+	This study

KGY829-2	*cps1-191 clp1-W113R ade6-M21X ura4-D18 leu1-32* h^-	This study
KGY750-2	*cps1-191 clp1-Q111L,W113R ade6-M21X ura4-D18 leu1-32* h^+	This study
KGY33-2	*clp1-mEGFP:kanMX6 sad1-mCherry:natMX6 mCherry-cdc15 ade6-M21X leu1-32 ura4-D18* h^+	This study
KGY170-2	*clp1-Q111L,W113R-mEGFP:kanMX6 sad1-mCherry:natMX6 mCherry-cdc15 ade6-M21X leu1-32 ura4-D18* h^+	This study
KGY490-2	*clp1-mEGFP:kanMX6 nuf2-mRFP::*$ura4^+$ *ade6-M210? ura4-D18 leu1-32 h?*	This study
KGY491-3	*clp1-Q111L W113R-mEGFP:kanMX6 nuf2-mRFP::ura4+ ade6-M210? ura4-D18 leu1-32 h?*	This study
**Figure 6**		
KGY46-2	*clp1-mEGFP:kanMX6 ade6-M210 leu1-32 ura4-D18* h^-	This study
KGY48-2	*clp1-Q111L,W113R-mEGFP:kanMX6 ade6-M210 ura4-D18 leu1-32* h^-	This study
**Figure 7**		
KGY246	*ura4-D18 ade6-M210 leu1-32* h^-	Lab stock
KGY33-2	*clp1-mEGFP:kanMX6 sad1-mCherry:natMX6 mCherry-cdc15 ade6-M21X leu1-32 ura4-D18* h^+	This study
KGY170-2	*clp1-Q111L,W113R-mEGFP:kanMX6 sad1-mCherry:natMX6 mCherry-cdc15 ade6-M21X leu1-32 ura4-D18* h^+	This study
KGY445-2	*clp1(1-372)-mEGFP:kanMX6 sad1-mCherry:natMX6 mCherry-cdc15 ade6-M21X leu1-32 ura4-D18* h^+	This study
KGY1927-2	*clp1(1-372)-Q111L,W113R-mEGFP:kanMX6 mCherry-cdc15 sad1-mCherry:natMX6 ade6-M21X ura4-D18 leu1-32 h?*	This study
KGY343-2	*nop2-mCherry:hphMX6 clp1(1-372)-mEGFP:kanMX6 sad1-mCherry:natMX6 mCherry-cdc15 ade6-M21X leu1-32 ura4-D18* h^+	This study
KGY346-2	*nop2-mCherry:hphMX6 clp1(1-372)-Q111L,W113R-mEGFP:kanMX6 mCherry-cdc15 sad1-mCherry:natMX6 ade6-M21X ura4-D18 leu1-32 h?*	This study
KGY17141	*cps1-191 ura4-D18 leu1-32 ade6-M210* h^+	Lab stock
KGY4791-2	*cps1-191 clp1Δ::ura4+ ura4-D18 ade6-M21X leu1-32 lys1-151* h^-	Lab stock
KGY493-2	*clp1(1-372)-mEGFP:kanMX6 cps1-191 ura4-D18 leu1-32 ade6-M210* h^+	This study
KGY498-2	*clp1(1-372)-Q111L,W113R-mEGFP:kanMX6 cps1-191 ade6-M21X ura4D-D18 leu1-32 h?*	This study
**Figure S1**		
KGY1162	*mid1-mNG:kanMX6 ade6-M210 ura4-D18 leu1-32* h^+	This study
KGY19945	*mid1-L448A-mNG:kanMX6 ade6-M21X ura4-D18 leu1-32* h^+	This study
**Figure S2**		
PJ69-4A	*MATa trpl-901 leu2-3,112 ura3-52 his3-200 ga14Δ ga180Δ LYS2::GALI-HIS3 GAL2-ADE2 met2::GAL7-lacZ*	Lab stock
KGY8749	*clp1-EGFP:kanMX6 leu1+::P3nmt1-nsk1-T.nmt1:leu1-32 ura4-D18 ade6-M210 leu1-32* h^-	This study
KGY7351	*nog1-mCherry:kanMX6 ura4-D18 ade6-M210 leu1-32* h^-	Lab stock
KGY16986-2	*nsk1-F168A ade6-M210 leu1-32 ura4-D18 h-*	This study
KGY5739	*alp7::*$ura4^+$ *ade6-M210 ura4-D18 leu1-32* h^+	Lab stock
KGY9504-2	*nsk1-F168A alp7::*$ura4^+$ *ade6-M210 leu1-32 ura4-D18 h?*	This study
KGY9580	*nsk1-linkerGFP:kanMX6 sid4-RFP:kanMX6 ade6-M21X ura4-D18 leu1-32* h^-	Lab stock

KGY447-2	*nsk1-F168A-linkerGFP:kanMX6 sid4-RFP:kanMX6 ade6-M21X leu1-32 ura4-D18 h?*	This study
**Figure S4**		
KGY246	*ura4-D18 ade6-M210 leu1-32* h^-	Lab stock
KGY223-2	*clp1Δ::kanMX6 ura4-D18 ade6-M210 leu1-32 h?*	Lab stock
KGY4587	*clp1-D257A ura4-D18 ade6-M210 leu1-32* h^-	Lab stock
KGY4972	*clp1-C286S ura4-D18 ade6-M210 leu1-32* h^-	Lab stock
KGY1-2	*clp1-Q111L ura4-D18 ade6-M210 leu1-32* h^+	This study
KGY19942	*clp1-W113R ura4-D18 ade6-M210 leu1-32* h^+	This study
KGY1421-2	*clp1-Q111L,W113R ade6-M21X ura4-D18 leu1-32* h^-	This study
KGY46-2	*clp1-mEGFP:kanMX6 ade6-M210 ura4-D18 leu1-32 h-*	This study
KGY48-2	*clp1-Q111L,W113R-mEGFP:kanMX6 ade6-M210 ura4-D18 leu1-32 h-*	This study
**Figure S5**		
KGY881-2	*clp1-EGFP:kanMX6 mCherry-Cdc15 sid4-RFP:kanMX6 ade6-M210 leu1-32 ura4-D18* h^+	This study
KGY562-2	*clp1-Q111L-EGFP:kanMX6 mCherry-cdc15 sad1-mCherry:natMX6 ade6-M210 leu1-32 ura4-D18* h^-	This study
KGY572-2	*clp1-W113R-EGFP:kanMX6 mCherry-cdc15 sad1-mCherry:natMX6 ade6-M210 leu1-32 ura4-D18* h^-	This study
KGY782-2	*clp1-Q111L,W113R-EGFP:kanMX6 nog1-mCherry:kanMX6 ade6-M21X ura4-D18 leu1-32* h^+	This study
KGY7444	*cut17-275 ura4-D18 leu1-32 ade6-M216* h^-	Lab stock
KGY10801	*clp1-EGFP:kanMX6 nog1-mCherry:kanMX6 ade6-M210 leu1-32 ura4-D18* $h^?$	Lab stock
**Figure S6**		
KGY246	*ura4-D18 ade6-M210 leu1-32* h^-	Lab stock
KGY46-2	*clp1-mEGFP:kanMX6 ade6-M210 ura4-D18 leu1-32 h-*	This study
KGY48-2	*clp1-Q111L,W113R-mEGFP:kanMX6 ade6-M210 ura4-D18 leu1-32 h-*	This study
KGY3338-2	*clp1-Y106A,M107A-mEGFP:kanMX6 ura4-D18 ade6-M216 leu-32* h^-	This study
KGY38-2	*clp1-Y106A,M107A,Q111L,W113R-mEGFP:kanMX6 ura4-D18 ade6-M216 leu-32* h^-	This study
